# Evaluation of the Functional Suitability of Carboxylate Chlorin *e*_6_ Derivatives for Use in Radionuclide Diagnostics

**DOI:** 10.3390/pharmaceutics18010023

**Published:** 2025-12-23

**Authors:** Mariia Larkina, Anastasia Demina, Nikita Suvorov, Petr Ostroverkhov, Evgenii Plotnikov, Ruslan Varvashenya, Vitalina Bodenko, Gleb Yanovich, Anastasia Prach, Viktor Pogorilyy, Sergey Tikhonov, Alexander Popov, Maxim Usachev, Beatrice Volel, Yuriy Vasil’ev, Mikhail Belousov, Mikhail Grin

**Affiliations:** 1 Department of Pharmaceutical Analysis, Siberian State Medical University, Moscow Tract, 2, 634050 Tomsk, Russia; marialarkina@mail.ru (M.L.); sonne_gleb@mail.ru (G.Y.); mvb63@mail.ru (M.B.); 2 Research Centrum for Oncotheranostics, Research School of Chemistry and Applied Biomedical Sciences, Tomsk Polytechnic University, Lenin Ave., 30, 634050 Tomsk, Russia; plotnikovev@tpu.ru (E.P.); mr.varvashenya@mail.ru (R.V.); bodenkovitalina@gmail.com (V.B.); nastya.prach@mail.ru (A.P.); 3 Department of Chemistry and Technology of Biologically Active Compounds, Medicinal and Organic Chemistry, Institute of Fine Chemical Technologies, MIREA—Russian Technological University, Vernadsky Ave., 86, 119571 Moscow, Russia; d.a.i00@mail.ru (A.D.); mrp_ost@mail.ru (P.O.); pogorilviktor@gmail.com (V.P.); deviantprince13th@gmail.com (S.T.); alexander.p.tmb@gmail.com (A.P.); maximus021989@mail.ru (M.U.); vasilev_yu_l@staff.sechenov.ru (Y.V.); michael_grin@mail.ru (M.G.); 4 Science and Education Laboratory for Chemical and Pharmaceutical Research, Siberian State Medical University, Moscow Tract, 2, 634050 Tomsk, Russia; 5 The Laboratory of Molecular Therapy of Cancer, Cancer Research Institute, Tomsk National Research Medical Center, Russian Academy of Sciences, Ushaika River Embankment, 10, 634009 Tomsk, Russia; 6 N.V. Sklifosovskyi Institute of Clinical Medicine, Rossolimo St., 11, Bld. 2, 119435 Moscow, Russia; beatrice.volel@gmail.com; 7 Department of Operative Surgery and Topographic Anatomy, I.M. Sechenov First Moscow State Medical University, Trubetskaya St., 8, Bld. 2, 119991 Moscow, Russia

**Keywords:** technetium-99m, chlorin, DTPA, radiopharmaceuticals, iminodiacetic acid, cancer

## Abstract

Radionuclide-based molecular imaging modalities are active and developing areas of functional and molecular diagnosis. Among the radionuclides used for SPECT imaging in oncology, ^99m^Tc is a leading candidate for radiolabeling. At present, a sufficient number of complexons for ^99m^Tc have been described; however, the development of effective delivery systems for this isotope to the area of interest is a complex research task. The use of tumor-targeting molecules as carriers for radioactive tracers is an effective strategy that has enabled the development of many novel radiopharmaceuticals for cancer imaging. **Background**: To date, a number of studies have shown tumorotropicity of tetrapyrrole compounds to tumor tissues, in particular derivatives of natural chlorophyll A. **Methods:** Purification was performed using solid-phase extraction. Assessment of radiochemical yield and purity was performed via radio-ITLC. The in vitro tumor cell accumulation was assessed using SKOV-3 and A-431 cell lines. Dose-dependent biodistribution was evaluated in Nu/J mice bearing epidermoid carcinoma (A-431) xenografts. **Results:** In this work, we obtained complexes with ^99m^Tc based on water-soluble carboxylate chlorin *e*_6_ derivatives in order to evaluate their potential for use as SPECT radiopharmaceuticals. We performed radiolabelling optimization of a series of the novel chlorins and primary preclinical studies, including an assessment of the effect of their lipophilicity and charge on tumor uptake. **Conclusions:** Modification of the periphery of the chlorin macrocycle with chelating groups allows for complexing a wide range of metals, including ^99m^Tc, which can be used for targeted delivery of the radionuclide to the area of interest.

## 1. Introduction

Radionuclide-based molecular imaging modalities are active and developing areas of functional and molecular diagnosis [1,2]. Specifically, single photon emission computed tomography (SPECT) is an attractive diagnostic technique due to its non-invasiveness, high diagnostic accuracy, whole body detection within one procedure, optimal availability, and widespread use [3,4,5]. Among the radionuclides for SPECT imaging, ^99m^Tc is a leading candidate for radiolabelling. The ^99m^Tc is characterized by an optimal nuclear decay characteristic (physical half-life of 6 h, which emits γ rays with energy of 140 keV (90%)), high spatial resolution, economical and readily available supply from ^99^Mo/^99m^Tc generator systems [6,7]. The use of tumor-targeting molecules as carriers for radioactive tracers is an effective strategy that has enabled the development of many novel radiopharmaceuticals for cancer imaging [8,9,10,11,12,13,14]. Both high-molecular compounds, which are proteins, and small target molecules, some of which are already used in clinical practice, can be used as vector molecules for delivering a radionuclide to the area of interest [15,16,17,18,19].

To date, a large number of studies have demonstrated the tropism of tetrapyrrole compounds to tumor tissues [20,21]. Such compounds include derivatives of natural and synthetic porphyrins, as well as their hydrogenated analogs. The main area of application of these compounds is photodynamic therapy of oncological diseases [22]. Today, there are three generations of such photosensitizers, differing in maximum light absorption and pharmacokinetic characteristics [23,24,25]. Of particular interest to researchers are derivatives of chlorophyll A, on the basis of which a number of second-generation photosensitizers used in clinical PDT [26,27,28,29] have been created. Derivatives of natural chlorins have absorption in the near IR region and a high quantum yield of singlet oxygen generation, as well as biocompatibility, low toxicity, and selectivity of accumulation in tumor tissues. Tumor selectivity is achieved primarily by the ability of tetrapyrrole compounds to bind to low-density lipoproteins (LDL). LDL operates as a «transporter» for chlorins into actively dividing cancer cells, which require cholesterol to build the cell membrane [30,31,32,33].

The presence of a pocket in the chlorin macrocycle allows chelation of a wide range of metal ions. Currently, a large number of complexes of chlorophyll A derivatives with metals have been obtained, which are promising as diagnostic and therapeutic agents in various fields of medicine [34,35,36,37]. The PDT method is successfully combined with various other therapeutic and diagnostic approaches in the fight against tumor diseases [38]. Such metal complexes are used as photosensitizers in photodynamic therapy, in magnetic resonance imaging as contrast agents, in enzyme models of bioorganic chemistry, etc. [39]. The introduction of a metal atom into the chlorin macrocycle leads to a significant change in the photophysical properties of the original photosensitizer, including absorption spectra, quantum yields of singlet oxygen generation or fluorescence. A number of metals, including radioactive ones, can expand the functionality of photosensitizers. However, the possibilities for introducing metals into the internal cavity of the chlorin macrocycle are limited due to the relatively small size of the coordination sphere and the distorted non-planar structure of the pigment molecules. Complexes of natural chlorins containing the ^99m^Tc atom in the macrocyclic cavity were previously obtained, but the described metal complexes had low stability [40,41].

To obtain various metal complexes, an approach can be used that involves introducing external chelating groups onto the periphery of chlorin molecules [34,35,36]. The therapeutic and diagnostic potential of metal complexes of chlorins of this type have been demonstrated in a number of studies [42,43,44,45,46]. We have previously synthesized a chlorin containing a hydrazinonicotinic acid residue in the pyrrole A macrocycle and also obtained a radiocomplex with ^99m^Tc based on it [47]. Despite high radiolabeling efficiency, [^99m^Tc]Tc-HYNIC-Chl exhibited limited tumor selectivity in A-431 xenograft-bearing Nu/j mice, with uptake values of only 0.5–1%ID/g across all tested doses. This is likely due to its high lipophilicity (logD = 1.24 ± 0.03) and a bulky modification of the technetium core with co-ligands. The in vitro and in vivo behavior of chlorin complexes is significantly impacted by lipophilicity, along with other molecular properties such as charge, size, and flexibility. It is plausible that adjusting these parameters may increase the tumor selectivity of the complex and lead to an improvement in the in vivo biodistribution profile

In this work, we obtained complexes with ^99m^Tc based on three water-soluble derivatives of chlorin *e*_6_ containing free carboxyl groups and tertiary nitrogen atoms at the periphery of the macrocycle, with the aim of assessing their potential for use as SPECT radiopharmaceuticals. We performed radiolabelling optimization of a series of the novel chlorins and primary preclinical studies of the best option. In vivo evaluations included selection of the optimal injected dose and the appropriate tumor xenograft model, and biodistribution over time.

## 2. Materials and Methods

### 2.1. Photosensitizers Preparation

^1^H NMR spectroscopic data were collected on a Bruker DPX300 instrument (Bruker Corporation, Billerica, MA, USA) operating at a frequency of 300 MHz. Spectra were recorded in deuterated dimethyl sulfoxide, and the residual solvent signal served as an internal reference for calibration.

LC-MS analysis was performed on a system comprising a Dionex UltiMate 3000 RS liquid chromatograph coupled to a Q-Exactive high-resolution hybrid mass spectrometer (both from Thermo Scientific, Waltham, MA, USA). The analysis employed a Pyramid C-18 reversed-phase column (Macherey-Nagel, Düren, Germany). The mobile phases were Milli-Q water (component A) and HPLC-grade isopropyl alcohol (component B; Carlo Erba, Val-de-Reuil, France), with the column temperature held at 40 °C. Samples (3.00 μL) were injected using an autosampler rinsed with HPLC-grade methanol (Fisher Chemical, Pittsburgh, PA, USA). MS detection utilized positive-ion heated electrospray ionization (HESI). The source was operated with a spray voltage of 4.0 kV, a capillary temperature of 200 °C, and an inlet capillary temperature of 350 °C. The nitrogen gas flows were set to 45 (nebulizing), 25 (auxiliary), and 5 (drying) arbitrary units. An S-lens RF level of 50 arbitrary units was applied. Full-scan mass spectra were recorded from *m*/*z* 350 to 2200 at a resolving power of 60,000.

**Figure 1:**
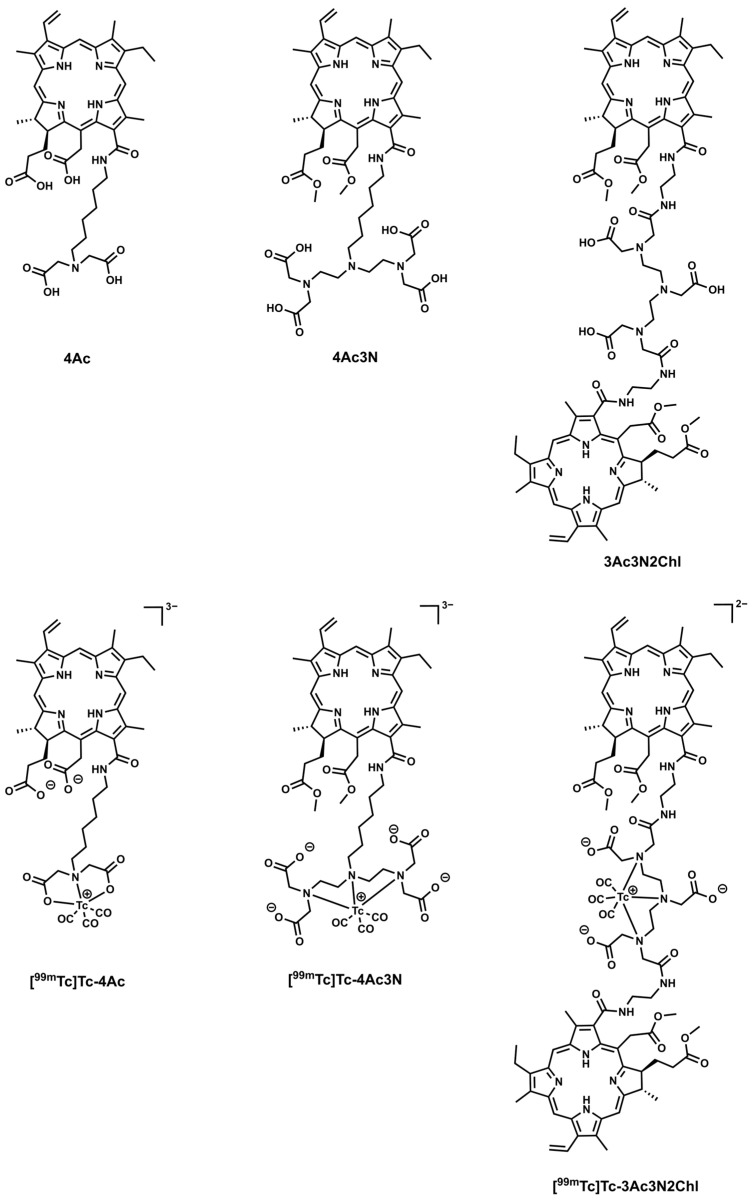
Structures of the tested ligands.

Photosensitizers 4Ac and 4Ac3N were prepared according to a previously described procedure (Figure 1) [43]. Photosensitizer 3Ac3N2Chl was prepared starting from aminoethylamide of chlorin *e*_6_ and DTPA anhydride using a previously described modified method [37]. To obtain compound 3Ac3N2Chl, 0.05 g (0.07 mmol) of aminoethylamide of methyl ester of pheophorbide *a* (Scheme S1) dissolved in 2 mL of DMF was added to 12.50 mg (0.035 mmol) of DTPA dianhydride, which was previously dissolved in 3 mL of DMF with the addition of 400 μL (2.87 mmol) of triethylamine. The reaction was carried out in a constant flow of argon at room temperature for 1.5 h. The obtained compound 3Ac3N2Chl was purified by column chromatography. (CH_2_Cl_2_/CH_3_OH; 11/1; *ν*/*ν*). Yield 0.025 g (50%). ^1^H NMR (300 MHz, DMSO-*d6*) δ ppm: 12.11 (3H, br s, COOH), 9.76 (2H, s, 10-H), 9.70 (2H, s, 5-H), 9.13 (2H, t, *J* = 5.1 Hz, 13^2^-NH), 9.09 (2H, s, 20-H), 8.26 (2H, dd, *J* = 17.8 Hz, 11.5 Hz, 3^1^-H), 8.12 (2H, t, *J* = 5.4 Hz, NH), 6.40 (2H, d, *J* = 17.8 Hz, E-3^2^-H), 6.13 (2H, d, *J* = 11.5 Hz, Z-3^2^-H), 5.49 (2H, d, *J* = 18.9 Hz, 15-CH_2_a), 5.31 (2H, d, *J* = 19.0 Hz, 15-CH_2_b), 4.62 (2H, q, *J* = 7.2 Hz, 18-H), 4.39 (2H, d, *J* = 9.5 Hz, 17-H), 3.79 (4H, m, 8^1^-CH_2_), 3.66 (6H, c, 15^3^-CO_2_CH_3_), 3.54 (6H, c, 17^4^-CO_2_CH_3_), 3.52–3.48 (26H, m, DTPA-CH_2_), 3.48 (12H, s, 2-CH_3_, 12-CH_3_), 3.27 (6H, s, 7-CH_3_), 2.68 (2H, m, 17^2^-CH_2_a), 2.32 (2H, m, 17^1^-CH_2_a), 2.13 (2H, m, 17^2^-CH_2_b), 1.84 (2H, m, 17^1^-CH_2_b), 1.62 (12H, m, 8^2^-CH_3_, 18-CH_3_), −1.83 (2H, br s, I—NH), −2.11 (2H, br s, III—NH). ESI-MS *m*/*z* calculated for C_90_H_111_N_15_O_18_ [M + H]^+^: 1691.97; [M + 2H]^2+^: 846.91. found: 1691.83; 846.42.

### 2.2. Radiolabelling Optimization

#### 2.2.1. Labelling Using Tin (II) Chloride as a Reducing Agent

Labelling of the tested chlorins was conducted using pre-prepared non-radioactive labeling compositions (kits 1, 2, 3, 4) containing tin (II) chloride as a reducing agent and excipients for reduction of ^99m^Tc (VII) ions. The formulations of the kits are shown in Table 1. For labelling, 100 µL (100–300 MBq) of ^99m^Tc-pertechnetate generator eluate ([^99m^Tc]TcO_4_^−^) and labelling kit (1, 2, 3 or 4) were added to 100–400 μg of 4Ac (2.4 mg/mL in Milli-Q water (Merck, Darmstadt, Germany)), 4Ac3N (3.6 mg/mL in Milli-Q water), or 3Ac3N2Chl (2.6 mg/mL in DMSO). The solutions obtained were shaken and incubated according to the conditions given in Table 1. Specifically, mixtures containing kits 1, 2, 3 were incubated at 60 °C for 60 min. To study the optimal conditions for labelling, the mixtures containing kit 4 were incubated by varying the temperature of incubation (60 °C, 70 °C, 85 °C) and the reaction time (30–60 min).

#### 2.2.2. Labelling Using Tricarbonyl Technetium-99m Precursor

The tricarbonyl technetium-99m precursor, ([^99m^Tc(CO)_3_(H_2_O)_3_]^+^), was prepared by adding 1000 μL (2–3 GBq) of generator-eluted ^99m^Tc-pertechnetate ([^99m^Tc]TcO_4_^−^) to a carbonyl reaction system (CRS) kit vial (PSI, Villigen, Switzerland). The mixture was incubated at 100 °C for 30 min. After incubation, 100 μL (200–400 MBq) of tricarbonyl technetium-99m precursor was added to 400 μg of 4Ac (2.4 mg/mL in Milli-Q water), 4Ac3N (3.6 mg/mL in Milli-Q water) or 3Ac3N2Chl (2.6 mg/mL in DMSO), followed by the addition of 100 µL of 0.1 M HCl. A pH range of 7.5 to 8.5 was established for the labeling. To evaluate the influence of temperature and reaction time on radiochemical yield of the ^99m^Tc-labeled complexes, the solutions obtained were shaken and incubated at 60 °C or 80 °C for 30 and 60 min.

Purification of the ^99m^Tc-chlorin complexes was performed using solid-phase extraction on a C18 Sep-Pak cartridge (360 mg, Waters Corp., Milford, MA, USA). Prior to sample application, the cartridge was conditioned with 10 mL each of 95% ethanol, 50% ethanol, and Milli-Q water. Free forms of radionuclide ions were eluted with 40 mL of Milli-Q water, followed by elution of the ^99m^Tc-labelled chlorins from the column with 2 mL of 95% ethanol solution.

The stability of ^99m^Tc-labelled chlorins in vitro was evaluated by incubation with a 1000-fold molar excess of histidine or PBS for 1, 2, 4, and 6 h. After incubation, the percentage of radioactivity released from the ^99m^Tc-labelled chlorin complex was measured by radio-ITLC.

Assessment of radiochemical yield and purity was performed via radio-instant thin-layer chromatography (radio-ITLC). Analyses were run on silica gel strips with the following eluents: mobile phase A (PBS) and mobile phase B (5 M ammonium acetate in methanol, 2:3 ratio). In system A, ^99m^Tc-labeled chlorins and the reduced hydrolyzed technetium colloid (RHT, [^99m^Tc]TcO_2_) remained at the application site (Rf  =  0.0), while free radionuclide and ^99m^Tc-labeled complexes with coligands moved with the solvent front (Rf  =  0.8–1.0). RHT levels were measured using system B, with ^99m^Tc-colloid remaining at the application site (Rf = 0.0), whereas other forms of ^99m^Tc ions and ^99m^Tc-labelled chlorins migrated with the solvent front (Rf = 0.9–1.0). Radiochemical purity was calculated by subtracting the percentage of RHT-associated activity determined in system B from the percentage at the site of application defined in system A. The distribution of radioactivity along the chromatographic plate was measured using a miniGITA Single iTLC scanner (Elysia Raytest, Straubenhardt, Germany).

To determine the Log D value, 10–15 µL (10 pmol) of the ^99m^Tc-chlorin complex was introduced into a 1:1 mixture of n-octanol and pH 7.4 Milli-Q water (500 µL each) contained within a LoBind tube (Eppendorf, Hamburg, Germany). The tube was vortexed for 3 min to ensure equilibration and then centrifuged for 5 min to achieve complete phase separation. Aliquots (100 μL) from each phase were transferred into tared vials, and their radioactive activity and weight were determined. This procedure was carried out in triplicate.

### 2.3. In Vitro Characterization

The in vitro tumor cell accumulation of the ^99m^Tc-labeled chlorins was assessed using SKOV-3 (ovarian adenocarcinoma) and A-431 (epidermoid carcinoma) cell lines (ATCC). The cells were cultured in Roswell Park Memorial Institute (RPMI)-1640 medium (Biowest, Nuaille, France). The complete medium contained 10% FBS (Sigma-Aldrich, St. Louis, MO, USA), 2 mM l-glutamine, and penicillin-streptomycin solution (Biowest, Nuaille, France).

For binding studies, cells were plated at a density of 7 × 10^5^ cells/dish and cultured for 24 h before incubation with the ^99m^Tc-labeled chlorins. For each time point, 3 Petri dishes were used for SKOV-3 and A-431 cell lines. The cells were incubated for 24 h at 37 °C, 5% CO_2_ to form a monolayer culture. Prior to the experiment, the media was removed, and the cells were washed with PBS. To select the optimum test concentration, the [^99m^Tc]Tc(CO)_3_-4Ac and [^99m^Tc]Tc(CO)_3_-4Ac3N chlorins (10, 100, and 1000 nM) in complete medium were simultaneously added to the dishes, followed by incubation for 1 h at 37 °C in 5% CO_2_. To assess the cell binding over time, the [^99m^Tc]Tc(CO)_3_-4Ac and [^99m^Tc]Tc(CO)_3_-4Ac3N chlorins (100 nM) in complete medium were simultaneously added to the dishes, followed by incubation for 1, 4, 6, and 24 h at 37 °C in 5% CO_2_. Two sets of control dishes were used as negative controls. The ^99m^Tc-labelled chlorin (100 nM) in complete medium was added to the first set of control dishes and incubated for 1 h at 4 °C to inhibit internalization. The inhibition of internalization was performed to establish the non-specific binding of chlorins to the surface of tumor cells. The same concentration of ^99m^Tc-labelled chlorin (100 nM) was added to second set of control dishes containing unprotected frozen or ethanol-treated dead tumor cells and incubated for 1 h at 37 °C in 5% CO_2_. In all groups of dishes tested, cell medium was collected after the required incubation period. The cells were then trypsinized, and the cell debris was collected, followed by a wash with PBS. Radioactivity of the medium and the cell fractions was measured with a PerkinElmer 2480 Wizard2 gamma spectrometer with a NaI (Tl) detector. From these measurements, the percentage of cell-associated activity was calculated.

### 2.4. In Vivo Characterization

Dose-dependent biodistribution of [^99m^Tc]Tc(CO)_3_-4Ac3N was evaluated in 12 female Nu/J mice bearing epidermoid carcinoma (A-431) xenografts. Xenografts were established by subcutaneous implantation of 10^7^ A-431 cells in 100 µL of media two weeks prior to the experiment. The average animal and tumor weights at the time of experiment were 25.4 ± 1.7 g and 0.41 ± 0.31 g, respectively. The mice were randomly assigned to one of three experimental groups (n = 4). To evaluate the tumor uptake and dose effect on biodistribution, mice bearing A-431 xenografts were injected intravenously into the tail vein with 1.2 mg/kg (30 µg/mouse, 60 kBq), 6 mg/kg (150 µg/mouse, 60 kBq), 12 mg/kg (300 µg/mouse, 60 kBq) of [^99m^Tc]Tc(CO)_3_-4Ac3N. The injected mass of [^99m^Tc]Tc(CO)_3_-4Ac3N (up to 30, 150, or 300 μg according to the injected dose) was adjusted by a corresponding unlabeled 4Ac3N. The final injection volume for each mouse was brought to 100 µL with PBS. The biodistribution was measured 2 h post-injection, which is a clinically relevant time point. The mice were euthanized under anesthesia by cervical dislocation, exsanguinated, and dissected. The radioactivity in weighed samples of blood and dissected organs was measured and standardized as the percentage of injected dose per gram of sample (%ID/g).

Biodistribution to select the optimal xenograft mouse model for the tested chlorin was performed in Nu/J mice with epidermoid carcinoma (A-431), prostate cancer (PC-3), and ovarian adenocarcinoma (SKOV-3) xenografts. To assess biodistribution in Nu/J mice with epidermoid carcinoma (A-431), a group with injection of 6 mg/kg (150 µg/mouse, 60 kBq) of [^99m^Tc]Tc(CO)_3_-4Ac3N from dose-dependent biodistribution was examined. For implantation of tumors, 10^7^ A-431, PC-3, or SKOV-3 cells in 100 µL of media were subcutaneously injected on the hind leg of female Nu/J mice. Four mice in each tumor xenograft model group were used. Xenografts were established two weeks prior to the experiment (one week for PC-3 xenografts). The average animal weight was 25.4 ± 1.7 g in the A-431 group, 24.6 ± 1.8 g in the PC-3 group, and 24.2 ± 2.7 g in the SKOV-3 group. The average tumor weight was 0.41 ± 0.31 g for A-431 tumor xenografts, 0.8 ± 0.2 g for PC-3 tumor xenografts, and 0.55 ± 0.12 g for SKOV-3 tumor xenografts. The mice were injected intravenously into the tail vein with 6 mg/kg (150 µg/mouse, 60 kBq) of [^99m^Tc]Tc(CO)_3_-4Ac3N. The injected mass of chlorin (up to 150 μg) was adjusted by a corresponding unlabelled chlorin 4Ac3N. The total injected volume was adjusted with PBS to 100 µL/mouse. The mice were euthanized under anesthesia by cervical dislocation, exsanguinated and, dissected at 2 h post-injection. The radioactivity in weighed samples of blood and dissected organs was measured and standardized as the percentage of injected dose per gram of sample (%ID/g).

The biodistribution over time was evaluated in 36 female Nu/J mice bearing prostate cancer (PC-3) xenografts. Xenografts were established by subcutaneous implantation of 10^7^ PC-3 cells in 100 µL of media one week prior to the experiment. The average animal and xenograft weights at the time of experiments were 25.1 ± 1.3 g and 0.6 ± 0.2 g, respectively. A group of four mice was used for each data point. Biodistribution was evaluated 1, 3, 6, 24, and 48 h post-injection. The mice were injected intravenously into the tail vein with 6 mg/kg (150 µg/mouse) [^99m^Tc]Tc(CO)_3_-4Ac3N. Depending on the time of dissection, the administered activity was from 60 to 5000 kBq. At each time-point, the animals were euthanized under anesthesia by cervical dislocation, exsanguinated, and dissected. The radioactivity in weighed samples of blood and dissected organs was measured and standardized as the percentage of injected dose per gram of sample (%ID/g).

## 3. Results

### 3.1. Synthesis and Radiolabelling of the Chlorins Complexes

As ligands for ^99m^Tc, we used molecules of natural chlorins containing different numbers of free carboxyl groups, as well as tertiary nitrogen atoms on the periphery of the macrocycle (Figure 1). Chlorin derivative containing an iminodiacetic acid residue (4Ac) was obtained starting from 13^1^-*N*-(aminohexyl)chlorin *e*_6_ dimethyl ester by the alkylation of the terminal amino group with bromomethyl acetate, followed by alkaline hydrolysis of the ester groups. Thus, we obtained a ligand containing four free carboxyl groups (two in positions 15 and 17 of the chlorin macrocycle, two in the imidiacetic residue), as well as one tertiary nitrogen atom. Derivative containing the same number of carboxyl groups, but three tertiary nitrogen atoms (4Ac3N) were obtained by a similar procedure by alkylation of 13^1^-*N*-(aminoethyl)chlorin *e*_6_ dimethyl ester with *N*,*N*-bis[(tert-butoxycarbonyl)methyl]-2-bromoethylamine, followed by acidolysis. We have already used 4Ac and 4Ac3N earlier as ytterbium complexons; their synthesis was described by us earlier [43]. In order to expand the range of ligands, we also obtained compound 3Ac3NChl containing two chlorin macrocycles in the complexone with three free carboxyl groups and three nitrogen atoms. The starting material for the synthesis of the 3Ac3NChl derivative was aminoethylamide of dimethyl ester of chlorin *e*_6_, which was treated with diethylenetriaminepentaacetic acid anhydride, which led to the formation of the desired product (Scheme S1). For the obtained ligands 4Ac, 4Ac3N, 3Ac3N2Chl, we studied their ability to form complexes with ^99m^Tc.

Two methods have been studied for the formation of radiocomplexes of chlorins 4Ac, 4Ac3N, 3Ac3N2Chl with technetium-99m. For labelling, tin (II) chloride with excipients (method 1) and production the tricarbonyl technetium-99m precursor (method 2) were used as the reducing agents for ^99m^Tc (VII) ions.

#### 3.1.1. Labelling Using Tin (II) Chloride as a Reducing Agent

In this method, tin (II) ions in the form of tin dichloride dihydrate (SnCl_2_∙2H_2_O) in combination with excipients were used to reduce ^99m^Tc (VII) ions and incorporate it into the structure of the studied chlorins. Pre-prepared kits including tin dichloride dihydrate and excipients such as sodium citrate, sodium gluconate, tetrasodium EDTA, and ascorbic acid were used for labelling. The influence of the composition of the reaction mixture, the temperature, and the incubation time of the labelling on the radiochemical yield of the ^99m^Tc-labelled chlorin complexes [^99m^Tc]Tc-4Ac, [^99m^Tc]Tc-4Ac3N, and [^99m^Tc]Tc-3Ac3N2Chl was investigated (Table 1). Of all the compounds studied, only compound 4Ac3N tended to form a radiocomplex with technetium-99m, with radiochemical yields ranging from 5 to 25%. The highest radiochemical yield for [^99m^Tc]Tc-4Ac3N was 24.4 ± 4.7%, with a RHT level of 36.3 ± 2.8% (kit 4).

#### 3.1.2. Labelling Using Tricarbonyl Technetium-99m Precursor

In this method, ^99m^Tc (VII) ions in the form of technetium generator eluate ([^99m^Tc]TcO_4_^−^) were initially reduced to ^99m^Tc (I) ions in the form of a tricarbonyl complex ([^99m^Tc(CO)_3_(H_2_O)_3_]^+^). The precursor produced was then used to radiolabel the tested chlorins. To determine the optimal incubation time and temperature for labelling, incubation was performed at 60 °C or 80 °C for 30 and 60 min. Radiochemical yields of ^99m^Tc-labelled chlorins ranged from 10 to 40%, with RHT levels not exceeding 1–1.4%. The highest radiochemical yield was observed for [^99m^Tc]Tc(CO)_3_-4Ac3N and was 37.5 ± 3.2% (Table 2). Increasing the incubation time and temperature decreased the radiochemical yield. Therefore, an incubation time of 30 min and an incubation temperature of 60 °C were determined as optimal conditions for labelling in this method. After purification on a C18 cartridge, the radiochemical purity of [^99m^Tc]Tc(CO)_3_-4Ac and [^99m^Tc]Tc(CO)_3_-4Ac3N was over 90% (Table 3). [^99m^Tc]Tc(CO)_3_-3Ac3N2Chl was of approximately 70% radiochemical purity after purification. Due to this moderate purity, it was not subjected to further in vitro or in vivo evaluations.

The in vitro stability test showed that [^99m^Tc]Tc(CO)_3_-4Ac3N and [^99m^Tc]Tc(CO)_3_-4Ac were stable for 1–6 h after the labelling procedure (Table 3, Figures S3–S10).

To assess the lipophilicity of chlorins radiocomplexes, we determined their distribution coefficient between water and n-octanol. The resulting logD values were 0.67 ± 0.7 and 1.12 ± 0.14 for [^99m^Tc]Tc(CO)_3_-4Ac and [^99m^Tc]Tc(CO)_3_-4Ac3N, respectively.

### 3.2. In Vitro Characterization

**Figure 2:**
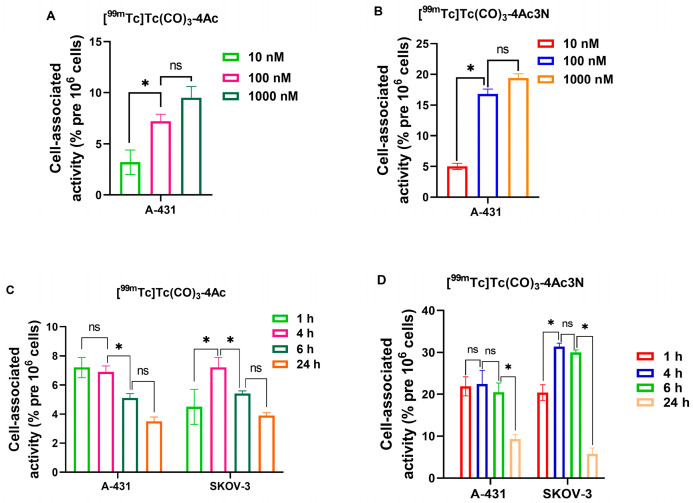
Results of cell-binding test after 1 h incubation with [^99m^Tc]Tc(CO)_3_-4Ac (**A**), [^99m^Tc]Tc(CO)_3_-4Ac3N (**B**) at a concentration of 10, 100, 1000 nM to A-431 cell line; after 1, 4, 6, 24 h incubation with [^99m^Tc]Tc(CO)_3_-4Ac (**C**), [^99m^Tc]Tc(CO)_3_-4Ac3N (**D**) at a concentration of 100 nM to A-431 and SKOV-3 cell lines. The data are presented as the average ± SD (n = 3). «*» indicates a significant difference between groups (*p* < 0.05, one-way ANOVA); «ns» indicates a non-significant difference (*p* < 0.05, one-way ANOVA).

The in vitro cell-binding assay for [^99m^Tc]Tc(CO)_3_-4Ac3N and [^99m^Tc]Tc(CO)_3_-4Ac was performed using A-431 (epidermoid carcinoma) and SKOV-3 (ovarian adenocarcinoma) cell lines. The effect of incubation time on cellular association was studied at four time points using a fixed concentration of the complexes [^99m^Tc]Tc(CO)_3_-4Ac3N and [^99m^Tc]Tc(CO)_3_-4Ac. The results of the in vitro experiments are displayed in Figure 2. The concentration of 100 nM of both ^99m^Tc-labelled chlorins provided a higher level of binding to A-431 cells compared to the concentration of 10 nM (Figure 2A,B). No significant differences were found for both chlorins tested at 100 nM compared to 1000 nM; therefore, a further cell binding assay was performed at the 100 nM chlorin concentration. Both ^99m^Tc-labelled chlorins showed a tendency to bind and accumulate in tumor cell lines tested. For each cell line investigated, the pattern of accumulation of the [^99m^Tc]Tc(CO)_3_-4Ac3N and the [^99m^Tc]Tc(CO)_3_-4Ac was similar. However, the [^99m^Tc]Tc(CO)_3_-4Ac variant showed remarkably low binding levels (with a maximum value of cell-associated activity of less than 8%) compared to [^99m^Tc]Tc(CO)_3_-4Ac3N for both cell lines. For A-431 cells, the percentage of cell-associated activity of the [^99m^Tc]Tc(CO)_3_-4Ac3N was observed to range from 20–23% after 1, 4, and 6 h of incubation, with no significant differences between time points (*p* > 0.05, one-way ANOVA). For the SKOV-3 cells, the accumulation was similar (*p* > 0.05, one-way ANOVA) after 1 h incubation, but significantly (*p* < 0.05, unpaired *t*-test) increased after 4 and 6 h of incubation to over 30%. Following 24 h of incubation, cell accumulation was significantly (*p* < 0.05, one-way ANOVA) reduced for both cell lines tested when compared to earlier time points. With regard to the control groups, both incubation with dead tumor cells and internalization inhibition demonstrated non-specific binding not exceeding 3%.

### 3.3. In Vivo Characterization

**Figure 3:**
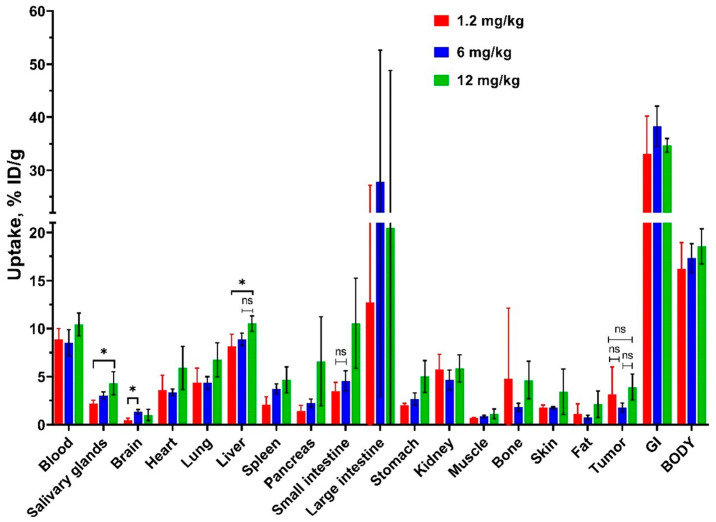
The dose-dependent biodistribution of [^99m^Tc]Tc(CO)_3_-4Ac3N in Nu/j mice bearing epidermoid carcinoma (A-431) xenografts at injected doses of 1.2 mg/kg, 6 mg/kg, 12 mg/kg, 2 h post-injection. The uptake is displayed as average %ID/g ± SD, except for the GI tract and body, where it is presented as average %ID/sample ± SD. «*» indicates a significant difference (*p* < 0.05, one-way ANOVA) between groups; «ns» indicates a non-significant difference (*p* < 0.05, one-way ANOVA).

Based on in vitro results, only the 4Ac3N variant was selected for further in vivo studies. The results of the evaluation of the effect of the injected doses of [^99m^Tc]Tc(CO)_3_-4Ac3N on the biodistribution in Nu/j mice bearing epidermoid carcinoma (A-431) xenografts are shown in Figure 3 and Table S1. The doses of 6 mg/kg and 12 mg/kg were considered to be close to the therapeutic dose (corrected for mice) for photodynamic therapy. A reduced dose of 1.2 mg/kg was also investigated. The biodistribution characteristics 2 h post-injection were similar for all doses tested, except for accumulation in certain healthy organs. The blood concentrations at this time point were elevated for all doses, exceeding 8.5%ID/g (*p* > 0.05, one-way ANOVA). Tumor uptake levels in epidermoid cancer xenografts were 3.2 ± 2.8%ID/g, 1.8 ± 0.5%ID/g, 3.9 ± 1.4%ID/g for doses of 1.2 mg/kg, 6 mg/kg, and 12 mg/kg, respectively. A non-significant difference was observed between tumor uptakes at any dose (*p* > 0.05, one-way ANOVA). The common feature observed for all doses examined was substantial uptake in the liver (averages above 8%ID/g), the large intestine with contents (averages above 13%ID/g), and residual GI organs (averages above 33%ID/g). The kidneys demonstrated slightly lower accumulation (averages above 5%ID/g), suggesting a predominant role for hepatobiliary excretion of chlorin. A noteworthy finding is the presence of low brain uptake rates (under 1.5%ID/g) for all doses.

Regarding differences in dose-dependent biodistribution, the administration of 12 mg/kg of chlorin resulted in a significantly higher level of small intestine uptake when compared to the other doses examined. The rate of liver uptake increased marginally but significantly (*p* > 0.05, one-way ANOVA) at the 12 mg/kg dose compared to the 6 mg/kg dose.

The uptakes in salivary glands and stomach are low, indicating a minimal release of free ^99m^Tc. Furthermore, the examined dose of 1.2 mg/kg of [^99m^Tc]Tc(CO)_3_-4Ac3N resulted in a significantly lower salivary glands uptake in comparison to the dose of 12 mg/kg (*p* < 0.05, one-way ANOVA).

**Figure 4:**
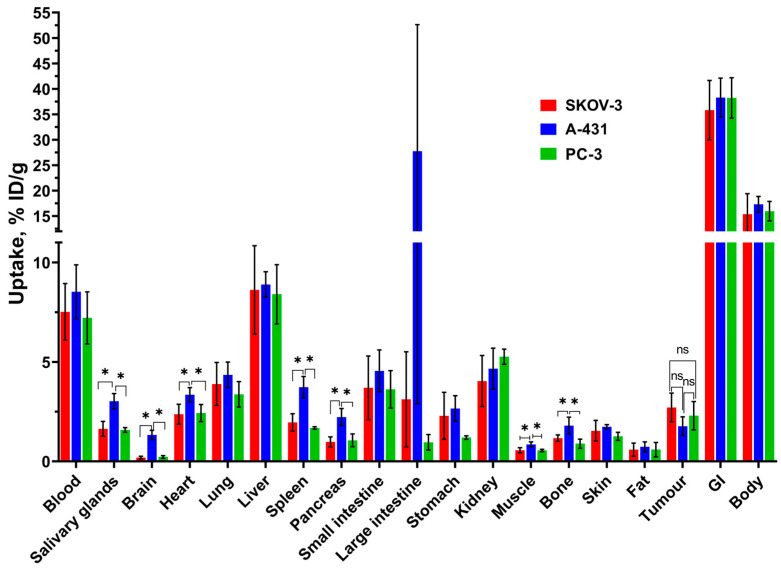
Biodistribution of [^99m^Tc]Tc(CO)_3_-4Ac3N in Nu/J mice with epidermoid carcinoma (A-431), prostate cancer (PC-3), and ovarian adenocarcinoma (SKOV-3) xenografts 2 h post-injection. The uptake is displayed as average %ID/g ± SD, except for the GI tract and body, where it is presented as average %ID/sample ± SD. «*» indicates a significant difference (*p* < 0.05, one-way ANOVA) between groups; «ns» indicates a non-significant difference (*p* < 0.05, one-way ANOVA).

The biodistribution data of [^99m^Tc]Tc(CO)_3_-4Ac3N (injected dose 6 mg/kg) in Nu/J mice bearing epidermoid carcinoma (A-431), prostate cancer (PC-3), and ovarian adenocarcinoma (SKOV-3) xenografts 2 h post-injection are shown in Figure 4 and Table S2. The tumor uptake on xenografts of the three cell lines tested showed no significant differences (1.8 ± 0.5%ID/g, 2.7 ± 0.7%ID/g, 2.3 ± 0.7%ID/g for A-431, SKOV-3, PC-3, respectively). However, variations in uptake were observed in healthy organs. For the mice bearing A-431 tumor xenografts, large intestine, salivary gland, cardiac, pancreatic, cerebral, muscle, and bone uptake levels are significantly higher compared to levels observed in mice with SKOV-3 and PC-3 tumor xenografts. No significant differences (*p* > 0.05) were found in the biodistribution of chlorin tested in the PC-3 and SKOV-3 xenograft mouse models.

**Figure 5:**
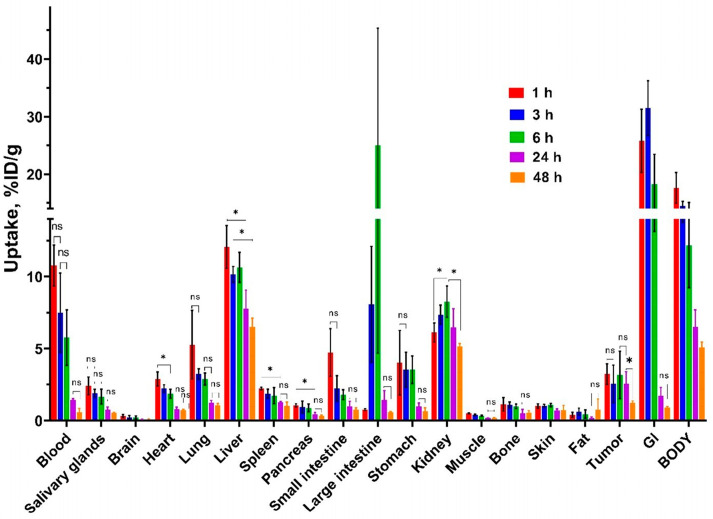
The biodistribution of [^99m^Tc]Tc(CO)_3_-4Ac3N in Nu/j mice bearing prostate cancer (PC-3) xenografts at 1, 3, 6, 24, and 48 h post-injection. The uptake is displayed as average %ID/g ± SD, except for the GI tract and body, where it is presented as average %ID/sample ± SD. «*» indicates a significant difference (*p* < 0.05, one-way ANOVA) between groups; «ns» indicates a non-significant difference (*p* < 0.05, one-way ANOVA).

The results of the [^99m^Tc]Tc(CO)_3_-4Ac3N biodistribution in Nu/j mice bearing prostate cancer (PC-3) xenografts over time (at 1, 3, 6, 24, and 48 h post-injection) are presented in Figure 5 and Table S3. Blood retention levels increased at the early time points (10.8 ± 1.4%ID/g at 1 h post-injection) and then gradually declined up to 48 h post-injection (0.6 ± 0.3%ID/g). No significant difference in tumor uptake was observed between 1 and 24 h post-injection; however, a statistically significant reduction occurred at 48 h post-injection.

Hepatic uptake was determined at a consistently high level (averages 6–12%ID/g) at 1–6 h post-injection (no significant difference, *p* > 0.05, between 1, 3, and 6 h post-injection) and slowly decreased further over time. The large intestine was noted for a significant escalation in accumulated activity (25.02 ± 20.33%ID/g) at 6 h post-injection. High levels of activity were also found in the rest of the gastrointestinal tract with contents at 1–6 h post injection (18–32%ID/g), followed by a sharp decline at 24 h post-injection. These data suggest substantial hepatobiliary excretion of the tested chlorin. Nevertheless, high renal absorption was also observed, with significantly (*p* < 0.05, one-way ANOVA) higher uptake at 3, 6 and 24 h post-injection compared to 1 h post-injection and a subsequent decrease (*p* < 0.05, one-way ANOVA) by 48 h post-injection. These data also indicate a significant involvement of renal excretion of chlorin.

In the lung, small intestine, and stomach, the [^99m^Tc]Tc(CO)_3_-4Ac3N uptake is 5%ID/g 1 h post-injection and decreases significantly with time (less than 1%ID/g 48 h after injection). The low uptake (under 2%ID/g) in the salivary glands and stomach after 1 h and the subsequent decrease in this uptake by 48 h (less than 1%ID/g) confirm the stability of the [^99m^Tc]Tc(CO)_3_-4Ac3N radiocomplex in vivo.

**Figure 6:**
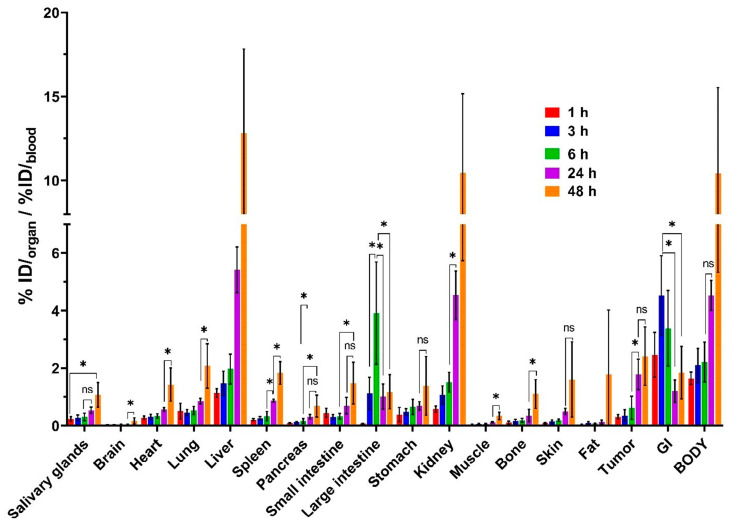
Comparison of organ-to-blood ratios from the biodistribution of [^99m^Tc]Tc(CO)_3_-4Ac3N in Nu/j mice bearing prostate cancer (PC-3) xenografts 1, 3, 6, 24, and 48 h post-injection. «*» indicates a significant difference (*p* < 0.05, one-way ANOVA) between groups; «ns» indicates a non-significant difference (*p* < 0.05, one-way ANOVA).

**Figure 7:**
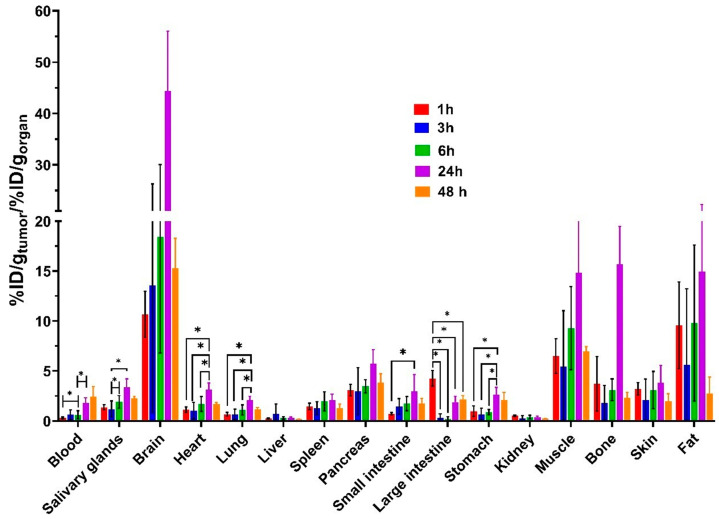
Comparison of tumor-to-organ ratios of [^99m^Tc]Tc(CO)_3_-4Ac3N in Nu/j mice bearing prostate cancer (PC-3) xenografts at 1, 3, 6, 24, and 48 h post-injection. «*» indicates a significant difference (*p* < 0.05, one-way ANOVA) between groups.

As illustrated in Figure 6 (Table S4), the organ-to-blood ratios is highest at 48 h post-injection in the main part of healthy organs, except tumor and organs of the GI tract, indicating intense uptake at the latest time point. The highest tumor-to-organ contrast for brain, pancreas, muscle, stomach, fat, skin, and bone, as shown in Figure 7 and Table S5, was observed at 24 h post injection. No significant differences (*p* > 0.05, one-way ANOVA) were found between the time points studied for tumor-to-skin, tumor-to-muscle ratios. The tumor-to-blood ratio was approximately two at 24 h after injection. The tumor-to-muscle ratio value was approximately six at 1 h after injection.

## 4. Discussion

Polydentate ligands are of particular interest for metal ion chelation in nuclear medicine due to their ability to adopt a fixed conformation in complex form [48]. Disodium diethylenetriaminepentaacetic acid (DTPA) is known as an effective chelating agent capable of forming stable complexes with various metal ions, such as calcium, zinc, ytterbium, lutetium, gadolinium, lanthanides, and with a number of radionuclides: ^99m^Tc, ^111^In, ^166^Ho, etc. [49,50]. Structural analogues of DTPA containing iminodiacetic acid residues are also used for radionuclide chelation and are contained in the ligands of some radiopharmaceuticals (Tc-Mebrofenin, Tc-DISIDA, Tc-PIPIDA) [51,52,53]. Previously, we described the synthesis and properties of water-soluble derivatives of chlorin *e*_6_ (4Ac and 4Ac3N) containing iminodiacetic acid and diethylenetriaminepentaacetic acid residues at the periphery of the macrocycle and studied their ability to complex lanthanide ions for use in fluorescent diagnostics (Figure 1) [43]. These derivatives can also be used to synthesize complexes with ^99m^Tc.

Also, the literature describes many examples of “cross-linked” DTPA derivatives with various organic substances, when two or more carboxyl groups of DTPA react, for example, with amino acids, hyaluronic acid, and polysaccharides [54,55,56,57,58]. Despite the fact that the “cross-linking” of DTPA leads to the fact that two of the five carboxyl groups cannot effectively participate in complexation, the three remaining ones, together with tertiary nitrogen atoms, are capable of forming fairly stable complexes with various metals [55], including radionuclides [59]. The advantage of such “cross-linked” derivatives is the simplicity of their synthesis by treating DTPA anhydride with nucleophilic reagents. In order to expand the range of chlorins studied, we also obtained such a cross-linked derivative 3Ac3N2Chl, containing two chlorin macrocycles in its composition.

Thus, we selected chlorins containing different amounts of tertiary nitrogen atoms and carboxyl groups on the periphery of the macrocycle. In this work, we studied the ability of the above-mentioned chlorins to form radiocomplexes with ^99m^Tc and assessed their functional suitability for use in radionuclide diagnostics.

Two approaches were employed for the labelling of chlorin complexes with technetium-99m. Using tin (II) dichloride dihydrate as a reducing agent for technetium-99m (VII) was the first approach [60]. This labelling method has been described for the chelating agents iminodiacetic acid and diethylenetriaminepentaacetic acid [61,62,63,64,65,66], fragments of which are present in the structure of chlorins studied. Specifically, tin (II) ions in the form of dihydrate dichloride, in combination with excipients such as sodium citrate, sodium gluconate, tetrasodium EDTA, ascorbic acid, were used to reduce ^99m^Tc (VII) ions of the technetium generator eluate (Na^99m^TcO_4_) and introduce it into the chlorin structure [67,68,69]. Excipients used can prevent undesirable processes occurring during ^99m^Tc reduction. In particular, excipients stabilize technetium complexes in intermediate oxidation state by preventing hydrolysis of the reducing agent to tin (IV) oxide SnO_2_ [65]. Critically low radiochemical yields and rather high levels of RHT were found to be the major drawbacks of this approach. Notably, of all the chlorins investigated, chlorin 4Ac3N was the only compound capable of forming a radiocomplex with technetium-99m under these conditions, but this complex was not stable.

The second approach was to reduce technetium-99m (VII) in the form of technetium generator eluate to technetium-99m (VII) in the form of tricarbonyl technetium precursor [69,70,71]. The disadvantage of this approach is the time-consuming labelling process [72]. However, this approach was successful and provided higher radiochemical yields compared to the first approach. The highest radiochemical yields of all tested chlorins were determined for 4Ac3N. Varying the labelling conditions, such as incubation time and temperature, did not significantly affect the radiochemical yields. Therefore, the lowest time and temperature were selected as optimal parameters for radiolabelling for subsequent in vitro and in vivo studies. The in vitro stability assessment using incubation with excess histidine as a competitive binding site for technetium-99m revealed high stability of the chlorin complex [^99m^Tc]Tc(CO)_3_-4Ac3N. The octanol–water distribution coefficient (Log D) indicated the high lipophilic nature of [^99m^Tc]Tc(CO)_3_-4Ac3N (1.12 ± 0.14). This agrees with the high lipophilicity of the previously studied [^99m^Tc]Tc-HYNIC-Chl (LogD = 1.24 ± 0.03) [47]. However, [^99m^Tc]Tc(CO)_3_-4Ac demonstrated lower lipophilicity (LogD = 0.67 ± 0.07) compared to the other complexes.

The in vitro cell binding assay showed that [^99m^Tc]Tc(CO)_3_-4Ac3N tended to accumulate sufficiently in both tumor cell lines tested. At the same time, [^99m^Tc]Tc(CO)_3_-4Ac showed low cell-associated levels and was not further considered for in vivo studies. A lipophilicity study showed that the complex was the most hydrophilic of all tested. The estimated charge of the studied complexes is identical, at −3. Probably, the differing lipophilicity of the complexes contributed to the variations in their cellular uptake levels. Thus, for these specific complexes, increased hydrophilicity correlated with reduced cellular accumulation. For each cell line investigated, the pattern of accumulation of both tested chlorins was similar. For the A-431 cell line, there were no significant differences after 1, 4, and 6 h of incubation. However, for the SKOV-3 cell line, the cellular accumulation of ^99m^Tc-labelled chlorins was significantly increased after 4 and 6 h of incubation, compared to the first time point for this cell line and compared to the level of accumulation in A-431 cells at these same time points. The different proliferation and metabolic rates in these cell lines may explain the dissimilar patterns of cellular retention of chlorin. The A-431 cell line is characterized by a higher rate of metabolic processes compared to SKOV-3, which is responsible for a more rapid internalization and subsequent wash-out of the chlorin radiocatabolites from the cells. After 24 h of incubation, the level of cell-associated activity was significantly reduced for both cell lines compared to earlier time points. This feature is characteristic of a non-residualizing label. Lipophilic radiometabolites of chlorins do not accumulate inside the cell after internalization but rapidly leak through the cell membrane. The non-residualizing label may lead to rapid elimination of the tested compound from non-targeted healthy organs. If the tested compound has a high affinity for tumor cells, the non-retained tag can improve the contrast of the diagnostic image.

For the in vivo characterization of the selected chlorin [^99m^Tc]Tc(CO)_3_-4Ac3N, we performed a series of in vivo studies, including dose-dependent biodistribution of the chlorin complex, comparison of its biodistribution in mice with different tumor xenograft models, and biodistribution over time (1–48 h post-injection) at the selected dose in the most appropriate tumor model.

For the dose-dependent biodistribution of [^99m^Tc]Tc(CO)_3_-4Ac3N in Nu/j mice bearing epidermoid carcinoma (A-431) xenografts, the doses of 6 mg/kg and 12 mg/kg (corrected for mice) were chosen to be close to the intended therapeutic doses. However, therapeutic doses of photosensitizers used clinically, particularly Chlorin *e*_6_, are quite high. In contrast, a lower dose can be used for a diagnostic agent compared to therapy. For this reason, it was also interesting to consider a reduced dose (1.2 mg/kg) and assess whether the reduction would affect biodistribution in the tumor and healthy organs. The results of dose-dependent biodistribution showed a common pattern for all doses examined, with slight differences in some healthy organs. Relatively high blood retention was observed for all doses tested. This blood retention is probably explained by the ability of chlorin to bind to blood proteins, including low-density lipoproteins [32,33,73]. This is a favorable property for prolonged kinetics in the blood, potentially increasing chlorins’ delivery to the tumor. However, with regard to the potential of 4Ac3N for radionuclide imaging with technetium-99m, the high retention in the blood 2 h post-injection shows that 4Ac3N does not have adequate imaging contrast at this time point. Regarding tumor uptake, ^99m^Tc-labelled 4Ac3N tended to accumulate in the epidermoid carcinoma with no significant differences at all doses investigated. A high uptake in the liver and in the organs of gastro-intestinal tract, especially in the contents of the large intestine, and a significant but somewhat lower uptake in the kidneys indicate a predominantly hepatobiliary elimination of the tested chlorin.

According to existing investigations, hepatobiliary excretion and high accumulation in the liver are characteristic of tetrapyrrole compounds [74], and of ^99m^Tc-labelled tetrapyrrole compounds in particular [75,76]. This is explained by the lipophilicity of the tetrapyrrole backbone, which increases tumor uptake but may contribute to the non-specific accumulation of tetrapyrrole compounds. By modifying chelate groups, biodistribution can be influenced to improve image contrast [6,76,77,78]. The main characteristics controlled are the hydrophilicity/lipophilicity of the radiolabelled complex chlorins [74]. Previous studies, however, have shown that [^99m^Tc]Tc-HYNIC-Chl exhibits nonspecific liver retention. This is attributed to its hydrophilic [^99m^Tc]Tc-HYNIC core and the coligands tricine and EDDA [47]. Another member of the tetrapyrrole group, the [^99m^Tc]Tc-HYNIC-porphyrin, in the study by Mohini Guleria et al. had similar aspects of biodistribution [75]. The charge, size, flexibility, and nature of functional groups also significantly impact the biodistribution of chlorins due to their small size of the molecules [34,78,79].

It is worth noting that in our study we investigated a chlorin radiocomplex based on tricarbonyl technetium-99m, which has a lipophilic character. Nevertheless, the presence of negative charges in the immediate vicinity may significantly reduce the effect of carbonyl technetium-99m on the liver uptake. The chelate group of 4Ac3N can be considered as a tridentate chelator for binding tricarbonyl technetium, and the carboxyl groups provide an additional negative charge. In this study, [^99m^Tc]Tc(CO)_3_-4Ac3N showed a three-fold lower uptake in the liver, higher tumor uptake, and lower uptakes in normal organs compared to [^99m^Tc]Tc-HYNIC-Chl, which we have previously investigated [47].

Interestingly, a low level of activity in the brain was observed, which demonstrates that certain amounts of ^99m^Tc-labelled 4Ac3N penetrate the blood–brain barrier (BBB). The progression of an aggressive neoplasm like the A-431 epidermoid carcinoma can induce systemic effects that disrupt BBB integrity [80,81]. This suggests the possible suitability of ^99m^Tc-labelled 4Ac3N for imaging brain tumors, which would be useful to examine in future research.

The in vivo stability of the [^99m^Tc]Tc(CO)_3_-4Ac3N complex is supported by its minimal accumulation in the salivary glands and stomach, where free ^99m^Tc formed by hydrolysis of the radiocomplexes can accumulate. The highest examined dose of 12 mg/kg resulted in increased hepatic and salivary gland uptake. For subsequent in vivo studies, a dose of 6 mg/kg was selected.

The biodistribution of [^99m^Tc]Tc(CO)_3_-4Ac3N in mice with different tumor xenograft models, specifically, in mice bearing epidermoid carcinoma (A-431), prostate cancer (PC-3), and ovarian adenocarcinoma (SKOV-3) xenografts 2 h post-injection showed no significant difference in tumor uptake. The selection of these models was deliberate and reflects the main mechanism of chlorin tumor targeting. Tetrapyrrole compounds, including tested derivatives, exploit the enhanced expression of low-density lipoprotein (LDL) receptors on rapidly proliferating cancer cells, which require increased cholesterol uptake for membrane biosynthesis [23,32,33]. This mechanism provides a degree of tumor selectivity that is independent of specific receptor expression characteristic of individual tumor types, thereby offering potential for broad-spectrum oncological imaging. The comparable tumor uptake observed across A-431, PC-3, and SKOV-3 xenografts (1.8–2.7%ID/g) supports this unspecific LDL-mediated targeting approach. We consider this ability as potential applicability for brain tumor imaging due to the potential penetration of the blood–brain barrier, warranting further investigation in appropriate glioma models. It is worth noting that most clinically approved radiopharmaceuticals rely on highly specific receptor or transporter targeting, e.g., PSMA, somatostatin receptors, or HER2. In this context, used chlorin-based approach occupies a unique niche between highly specific receptor-targeted radiopharmaceutical agents and fully metabolic tracers like ^18^F-FDG. While receptor-targeted radiopharmaceuticals offer superior tumor-to-background ratios in receptor-positive malignancies, they are inherently limited to tumors expressing a certain receptor.

Some variations in uptake were noted in healthy organs. For the mice bearing epidermoid carcinoma xenografts, large intestine, salivary gland, cardiac, pancreatic, cerebral, muscle, and bone uptake levels are significantly higher compared to levels observed in mice with ovarian adenocarcinoma and prostate cancer xenografts. The difference in biodistribution in mice with different tumor models can be explained by differences in tumor biochemistry, which affects blood vessel permeability, lymphatic drainage [82,83], low-density lipoprotein receptor expression on tumor cells [84] and on tumor vessel endothelial cells [85,86,87], macrophage numbers, and extracellular pH [88,89,90].

Based on the data from the previous in vivo stages, biodistribution assessments over time (1, 3, 6, 24, and 48 h post injection) were performed in mice bearing prostate cancer xenografts at an injected dose of 6 mg/kg of [^99m^Tc]Tc(CO)_3_-4Ac3N. The results of the biodistribution over time revealed that blood retention of the tested chlorin increased at early time points and then gradually decreased up to 48 h post-injection. Tumor uptake was not significantly different at 1–24 h post-injection but decreased significantly at 48 h post-injection. The results of the biodistribution over time also confirmed that the kidneys and liver intensively accumulate the radiocomplex and excrete it from the body. This should be considered to control potential nephro- and hepatotoxicity when injecting high-activity radiocomplexes. Additionally, the GI tract during the first 6 h is characterized by high accumulation of radiocomplex due to hepatobiliary circulation, which will reduce imaging contrast in this area.

The tumor-to-organ increased moderately from 1 to 24 h post-injection for all organs, except liver, and kidney. As we established, chlorin derivative provides moderate tumor uptake with favorable tumor-to-muscle ratios (>5:1) at all time points, comparable to early-generation ^99m^Tc-labeled peptides [91,92]. The highest ratios were tumor-to-brain, tumor-to-muscle, tumor-to-bone, and tumor-to-fat at 24 h pi. The tumor-to-organ suggests that the optimal time for high contrast imaging is 24–48 h. The half-life of technetium-99m is 6 h; therefore, this radionuclide is not suitable for imaging 48 h after injection. Indium-111, with a half-life of 2.7 days, may be more appropriate for this chlorin variant.

## 5. Conclusions

Thus, based on the radiochemical and biological studies carried out in this work, the radiocomplex [^99m^Tc]Tc(CO)_3_-4Ac3N was selected as the leader compound, which showed high stability and relatively high tropism to tumors of various genesis, which, in turn, determines its potential for radiolabeling in the diagnosis of oncological diseases. Increasing the efficiency of the leader radiocomplex in the future can apparently be achieved by changing the hydrophobic–hydrophilic balance of the latter by optimizing its structure, including the introduction of a chelating fragment into pyrrole A of the chlorin macrocycle, while maintaining the free DDTA fragment in the lower part of the molecule. Labeled 3Ac3N2Chl had low radiochemical purity due to complex instability. Labeled 4Ac was characterized by the highest hydrophilicity and lower cellular accumulation.

## Figures and Tables

**Table 1:** Composition of labelling kits, labelling conditions, and radiochemical yields for [^99m^Tc]Tc-4Ac, [^99m^Tc]Tc-4Ac3N, [^99m^Tc]Tc-3Ac3N2Chl chlorin radiocomplexes.

Kit	Kit Components	Labelling Conditions		Compound	Radiochemical Yield, %	
		Incubation Temperature, °C	Incubation Time, min		^99m^Tc-Labelled Chlorin + RHT (System A)	RHT (System B)
1	Tin (II) chloride (75 µg, 0.01 M in HCl, Fluka Chemika, Switzerland); sodium gluconate (5 mg, 50 mg/mL in Milli-Q water)	60	60	[^99m^Tc]Tc-4Ac	1.0 ± 0.1	2.6 ± 0.3
				[^99m^Tc]Tc-4Ac3N	1.6 ± 0.3	1.1 ± 0.1
				[^99m^Tc]Tc-3Ac3N2Chl	0.6 ± 0.2	0.7 ±0.2
2	Tin (II) chloride (75 μg, 0.01 M in HCl); sodium gluconate (5 mg, 50 mg/mL in Milli-Q water); tetrasodium EDTA (100 μg, 1 mg/mL in PBS)			[^99m^Tc]Tc-4Ac	92.3 ± 5.4	80.8 ± 4.2
				[^99m^Tc]Tc-4Ac3N	96.2 ± 4.2	76.4 ± 5.1
				[^99m^Tc]Tc-3Ac3N2Chl	57.4 ± 3.8	94.2 ± 5.2
3	Tin (II) chloride (50 µg, 4 mg/mL in 99.0% ethanol); ascorbic acid (25 µg, 2 mg/mL)			[^99m^Tc]Tc-4Ac	23.0 ± 3.1	12.4 ± 3.7
				[^99m^Tc]Tc-4Ac3N	65.0 ± 5.2	40.3 ± 5.6
				[^99m^Tc]Tc-3Ac3N2Chl	63.0 ± 1.6	64.3 ± 2.4
4	Tin (II) chloride (75 µg, 4 mg/mL in 0.1 M HCl); trisodium citrate (5 µL, 0.1 M)	60	60	[^99m^Tc]Tc-4Ac	19.6 ± 3.5	92.3 ± 1.1
				[^99m^Tc]Tc-4Ac3N	32.2 ± 4.2	22.3 ± 2.1
				[^99m^Tc]Tc-3Ac3N2Chl	22.2 ± 1.4	18.3 ± 3.5
		85	60	[^99m^Tc]Tc-4Ac	6.1 ± 0.5	16.9 ± 5.4
				[^99m^Tc]Tc-4Ac3N	9.7 ± 2.6	22.7 ± 2.3
				[^99m^Tc]Tc-3Ac3N2Chl	18.3 ± 1.9	39.3 ± 1.0
		70	30	[^99m^Tc]Tc-4Ac	6.3 ± 2.0	8.7 ± 0.5
				[^99m^Tc]Tc-4Ac3N	60.7 ± 4.2	36.3 ± 2.8
				[^99m^Tc]Tc-3Ac3N2Chl	2.9 ± 0.4	40.4 ± 1.5

**Table 2:** Labelling conditions and radiochemical yields for [^99m^Tc]Tc(CO)_3_-4Ac, [^99m^Tc]Tc(CO)_3_-4Ac3N, [^99m^Tc]Tc(CO)_3_-3Ac3N2Chl chlorin radiocomplexes.

Labelling Conditions		Radiochemical Yield, %		
Temperature, ◦C	Time, min	[^99m^Tc]Tc(CO)_3_-4Ac	[^99m^Tc]Tc(CO)_3_-4Ac3N	[^99m^Tc]Tc(CO)_3_-3Ac3N2Chl
60	30	27.3 ± 5.7	37.5 ± 3.2	19.3 ± 4.6
	60	25.8 ± 3.7	27.3 ± 5.7	15.1 ± 3.4
80	30	7.5 ± 2.8	10.3 ± 2.4	9.7 ± 3.2
	60	5.6 ± 1.4	7.3 ± 2.3	3.1 ± 0.8

**Table 3:** Radiochemical purity and results of the in vitro stability test for [^99m^Tc]Tc(CO)_3_-4Ac and [^99m^Tc]Tc(CO)_3_-4Ac3N chlorin radiocomplexes.

Radiocomplex	Conditions		Radiochemical Purity, %			
		After Labelling	After the In Vitro Stability Test			
			1 h	2 h	4 h	6 h
[^99m^Tc]Tc(CO)_3_-4Ac	1000-fold molar excess of histidine	93 ± 1	93 ± 1	93 ± 1	92 ± 0.5	91 ± 2
	PBS		92 ± 1.2	92 ± 1	91 ± 1	90 ± 1.5
[^99m^Tc]Tc(CO)_3_-4Ac3N	1000-fold molar excess of histidine	94 ± 1	94 ± 0.7	92 ± 1	91 ± 1	90 ± 2
	PBS		91 ± 1	91 ± 1.5	90 ± 1	90 ± 2

## Data Availability

Data is contained within the article or Supplementary Materials.

## Supplementary Materials

The following supporting information can be downloaded at: https://www.mdpi.com/article/10.3390/pharmaceutics18010023/s1, Scheme S1: Synthesis of 3Ac3N2Chl; Figure S1: Mass-spectrum of compound 3Ac3N2Chl; Figure S2: ^1^H NMR Spectrum of compound 3Ac3N2Chl; Figures S3–S10: Radio-iTLC chromatograms; Tables S1–S5: Biodistribution of [^99m^Tc]Tc(CO)_3_-4Ac3N.
