# Supplementary material for: Evaluation of the Functional Suitability of Carboxylate Chlorin e6 Derivatives for Use in Radionuclide Diagnostics
Source: Pharmaceutics. 2025 Dec 23;18(1):23. doi: 10.3390/pharmaceutics18010023 (PMC12844883; doi:10.3390/pharmaceutics18010023)
Supplement: Supplementary file 1 [file pharmaceutics-18-00023-s001.zip › pharmaceutics-4034875-supplementary.pdf]

# Evaluation of the Functional Suitability of Carboxylate Chlorin $e_6$ Derivatives for Use in Radionuclide Diagnostics

**Mariia Larkina [1,2], Anastasia Demina [3], Nikita Suvorov [3,*], Petr Ostroverkhov [3], Evgenii Plotnikov [2], Ruslan Varvashenya [2,4], Vitalina Bodenko [2,4], Gleb Yanovich [1,2], Anastasia Prach [2,5], Viktor Pogorilyy [3], Sergey Tikhonov [3], Alexander Popov [3], Maxim Usachev [3], Beatrice Volel [6,7], Yuriy Vasil'ev [3,7], Mikhail Belousov [1,2] and Mikhail Grin [3]**

1 Department of Pharmaceutical Analysis, Siberian State Medical University, Moscow Tract, 2, 634050 Tomsk, Russia; marialarkina@mail.ru (M.L.); sonne_gleb@mail.ru (G.Y.); mvb63@mail.ru (M.B.)
2 Research Centrum for Oncotheranostics, Research School of Chemistry and Applied Biomedical Sciences, Tomsk Polytechnic University, Lenin Ave., 30, 634050 Tomsk, Russia; plotnikovev@tpu.ru (E.P.); mr.varvashenya@mail.ru (R.V.); bodenkovitalina@gmail.com (V.B.); nastya.prach@mail.ru (A.P.)
3 Department of Chemistry and Technology of Biologically Active Compounds, Medicinal and Organic Chemistry, Institute of Fine Chemical Technologies, MIREA—Russian Technological University, Vernadsky Ave., 86, 119571 Moscow, Russia; d.a.i00@mail.ru (A.D.); mrp_ost@mail.ru (P.O.); pogorilviktor@gmail.com (V.P.); deviantprince13th@gmail.com (S.T.); alexander.p.tmb@gmail.com (A.P.); maximus021989@mail.ru (M.U.); vasilev_yu_l@staff.sechenov.ru (Y.V.); michael_grin@mail.ru (M.G.)
4 Science and Education Laboratory for Chemical and Pharmaceutical Research, Siberian State Medical University, Moscow Tract, 2, 634050 Tomsk, Russia
5 The Laboratory of Molecular Therapy of Cancer, Cancer Research Institute, Tomsk National Research Medical Center, Russian Academy of Sciences, Ushaika River Embankment, 10, 634009 Tomsk, Russia
6 N.V. Sklifosovskyi Institute of Clinical Medicine, Rossolimo St., 11, Bld. 2, 119435 Moscow, Russia; beatrice.volel@gmail.com
7 Department of Operative Surgery and Topographic Anatomy, I.M. Sechenov First Moscow State Medical University, Trubetskaya St., 8, Bld. 2, 119991 Moscow, Russia
* Correspondence: suvorov.nv@gmail.com; Tel.: +7-(915)756-37-31

## Supplementary Materials

**Contents**

**Synthesis of 3Ac3N2Chl** .......... 2
**Radio-iTLC chromatograms** .......... 4
**Biodistribution of [99mTc]Tc(CO)3-4Ac3N** .......... 8

**Synthesis of 3Ac3N2Chl**

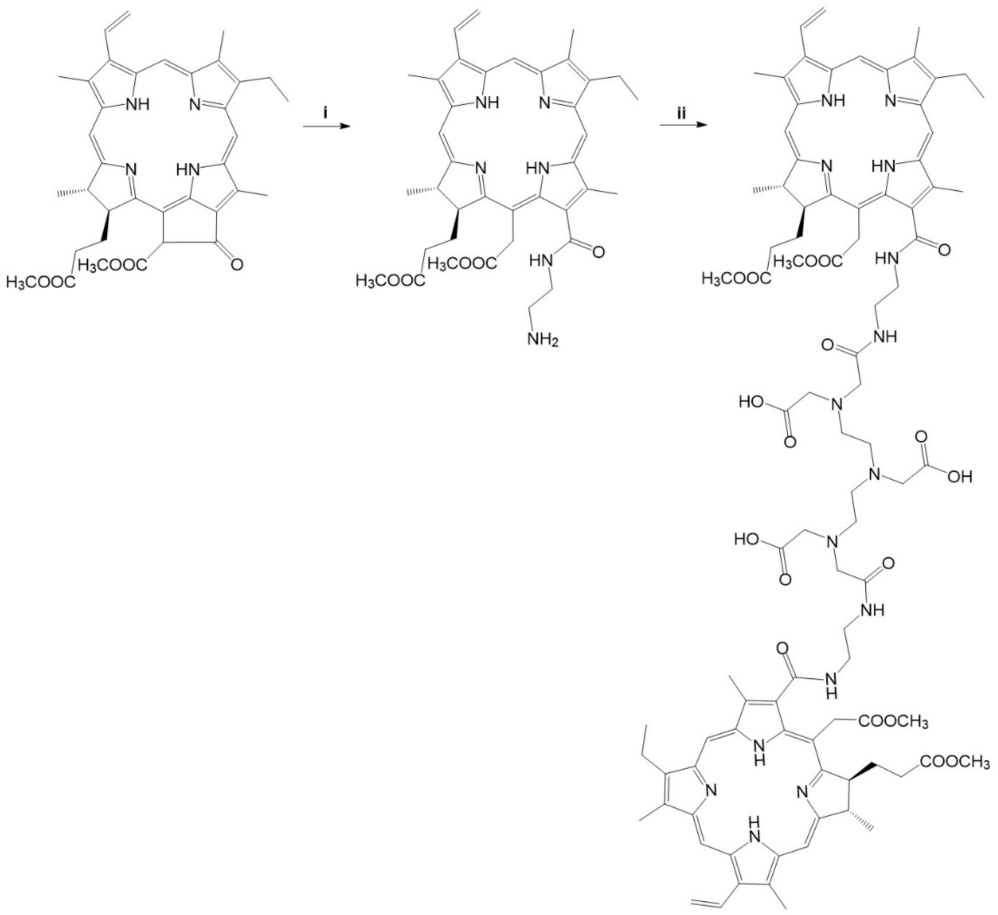


Scheme S1. Reagents and conditions: **i** – $NH_2(CH_2)_2NH_2$, TEA, DCM, 12 h, rt; **ii** – DTPA anhydride, TEA, DMF, 1.5 h, rt.

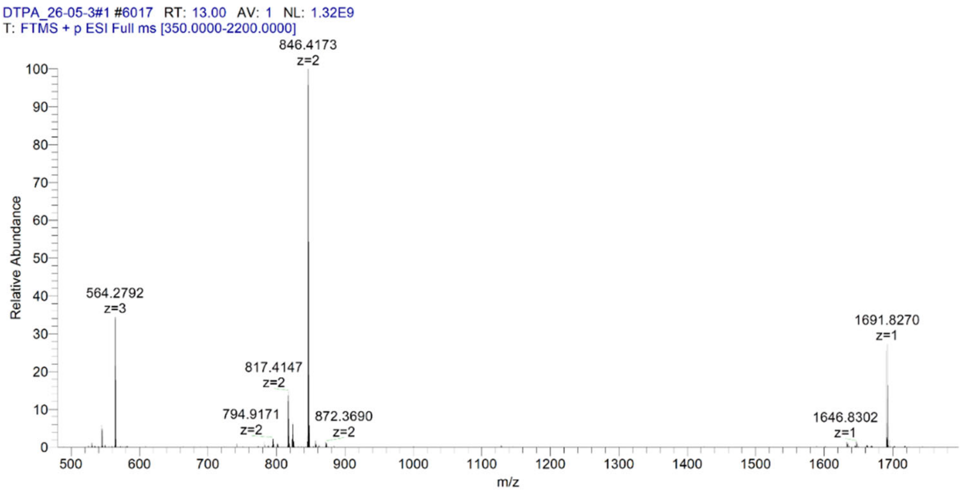


*Figure S1. Mass-spectrum (ESI/FT) of compound 3Ac3N2Chl*

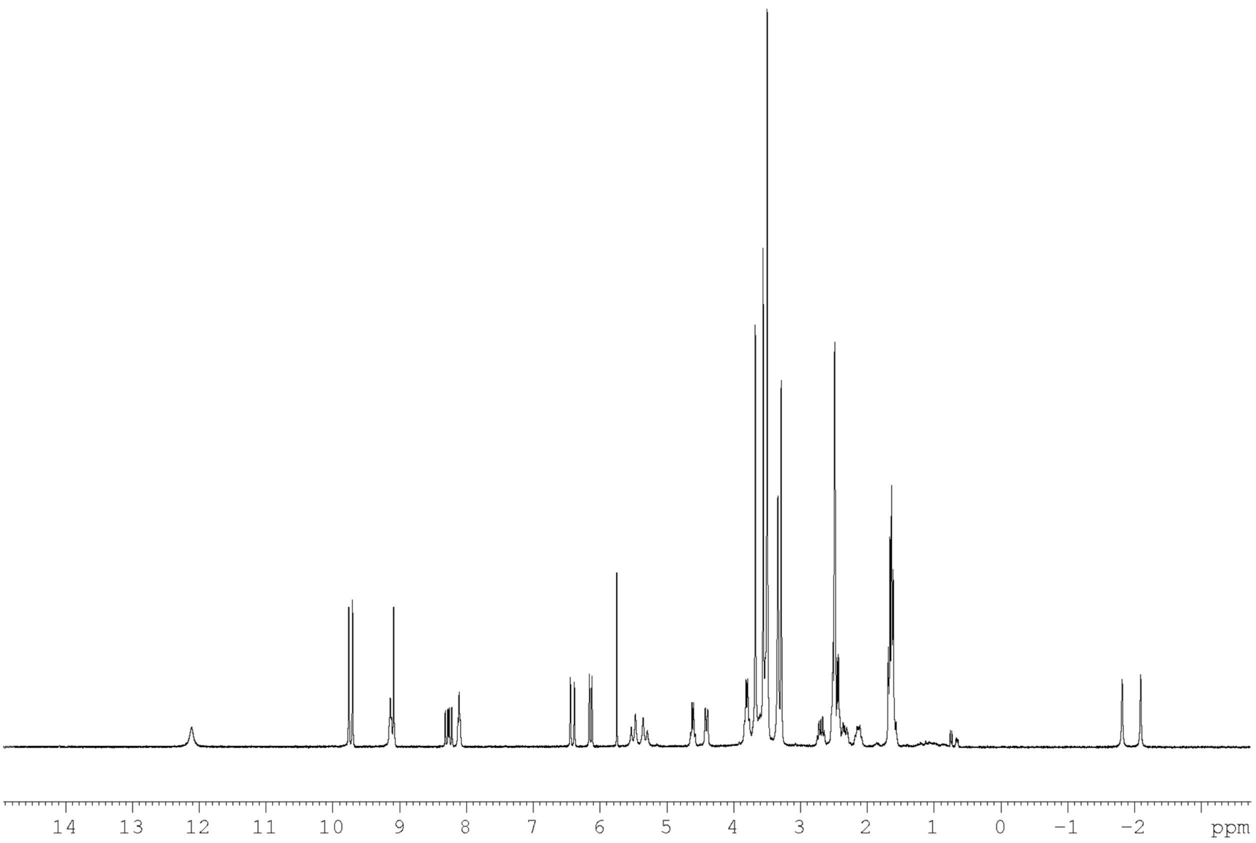


*Figure S2. $^{1}$H NMR Spectrum of compound 3Ac3N2Chl in DMSO-d6*

## Radio-iTLC chromatograms

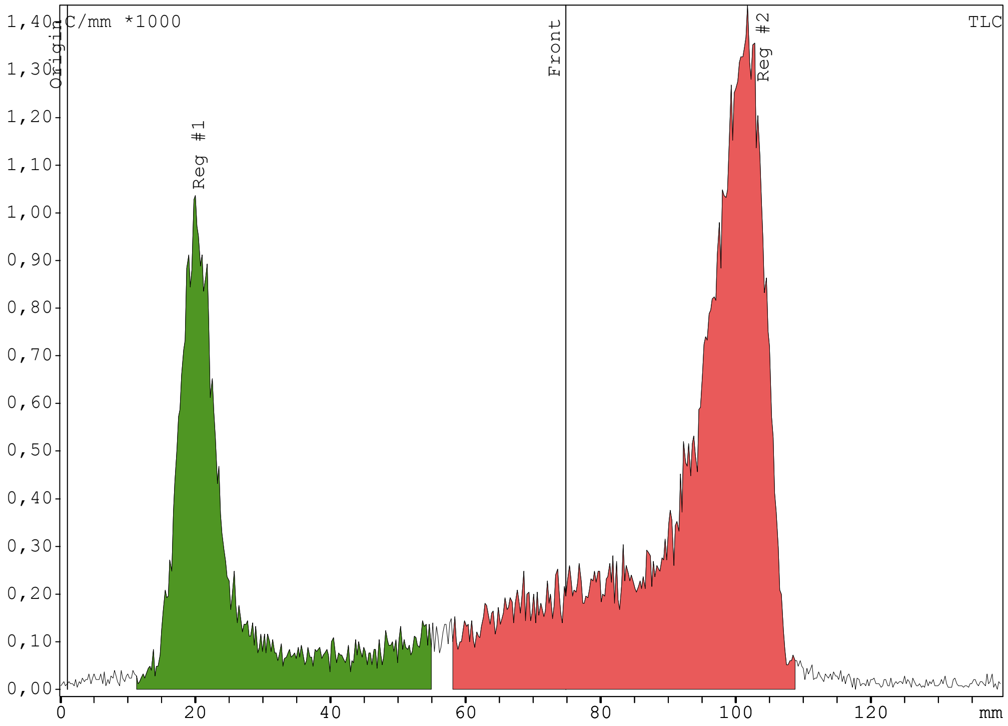


*Figure S3. Radio-iTLC chromatogram. Radiochemical yield of [$^{99m}$Tc]Tc(CO)$_3$-4Ac*

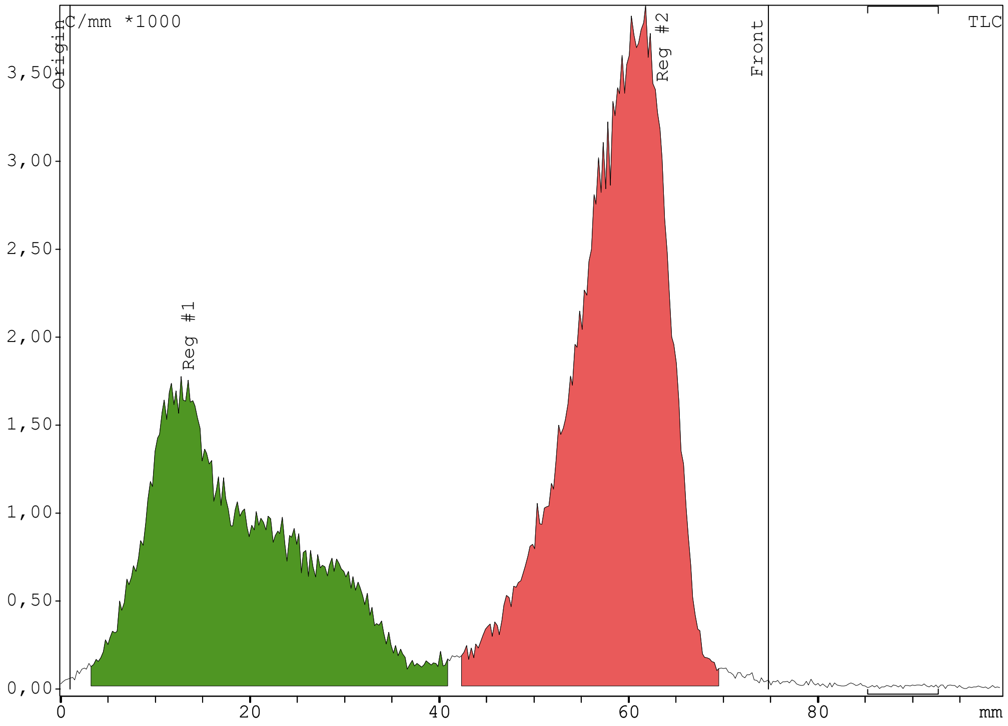


*Figure S4. Radio-iTLC chromatogram. Radiochemical yield of [$^{99m}$Tc]Tc(CO)$_3$-4Ac3N*

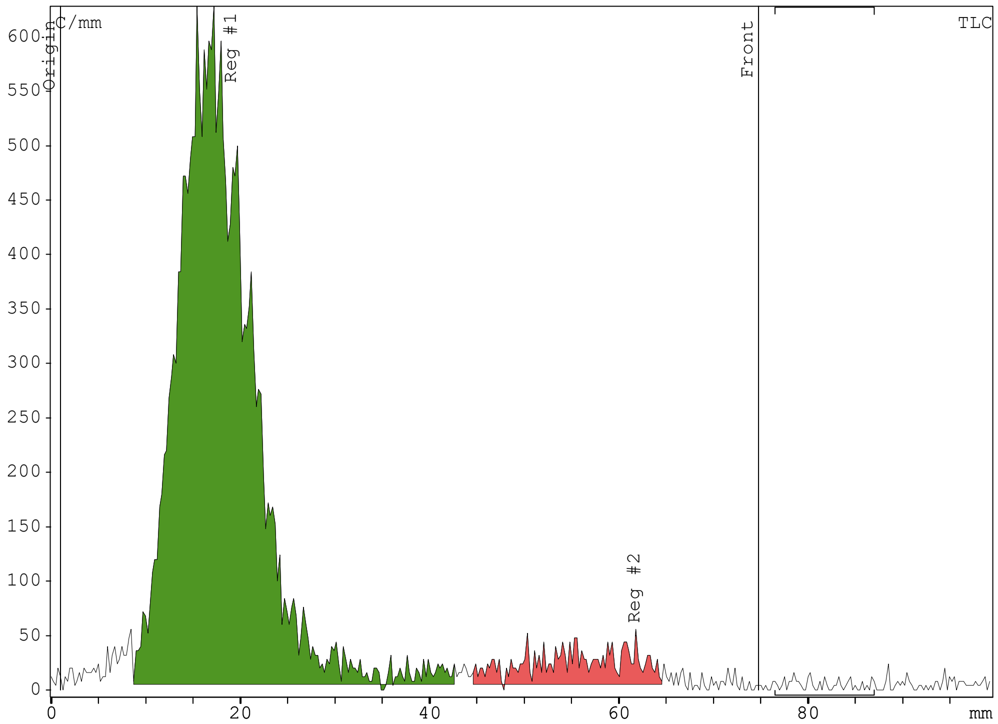


*Figure S5. Radio-iTLC chromatogram. Radiochemical purity of [$^{99m}$Tc]Tc(CO)$_3$-4Ac*

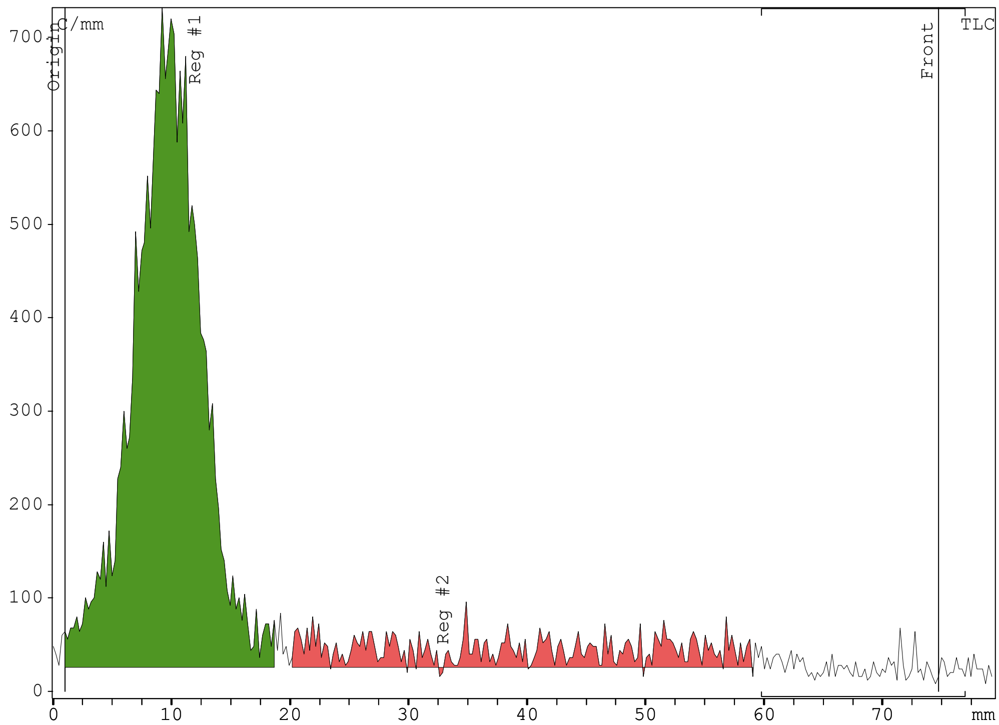


*Figure S6. Radio-iTLC chromatogram. Radiochemical purity of [$^{99m}$Tc]Tc(CO)$_3$-4Ac3N*

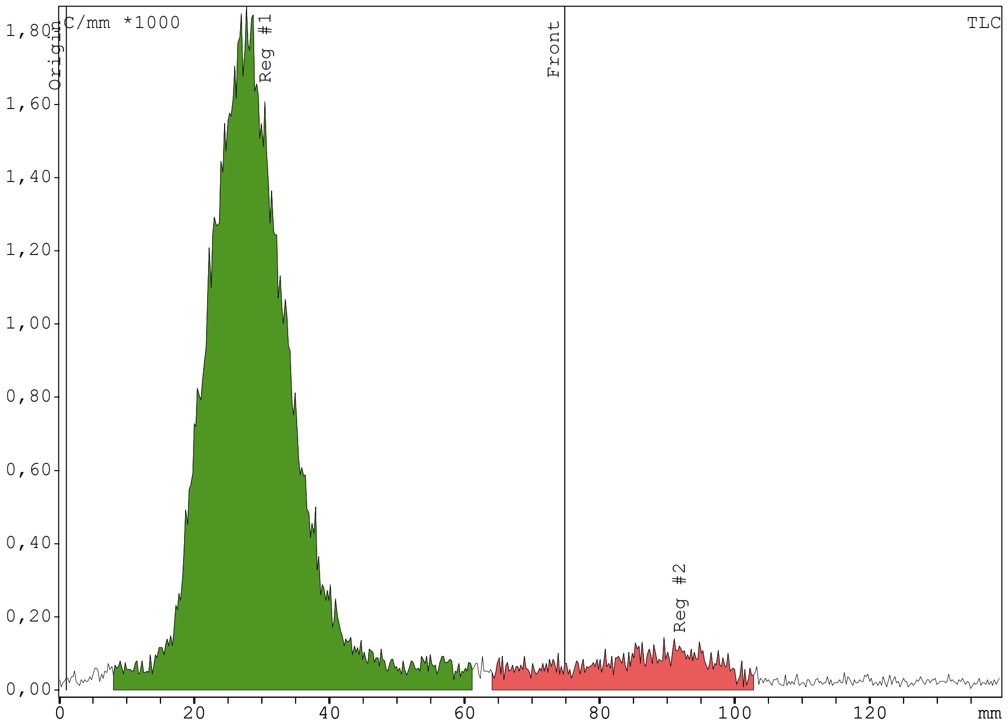


*Figure S7. Radio-iTLC chromatogram. Radiochemical yield of [$^{99m}$Tc]Tc-4Ac after 4Ac after incubation with PBS for 4 h (stability test)*

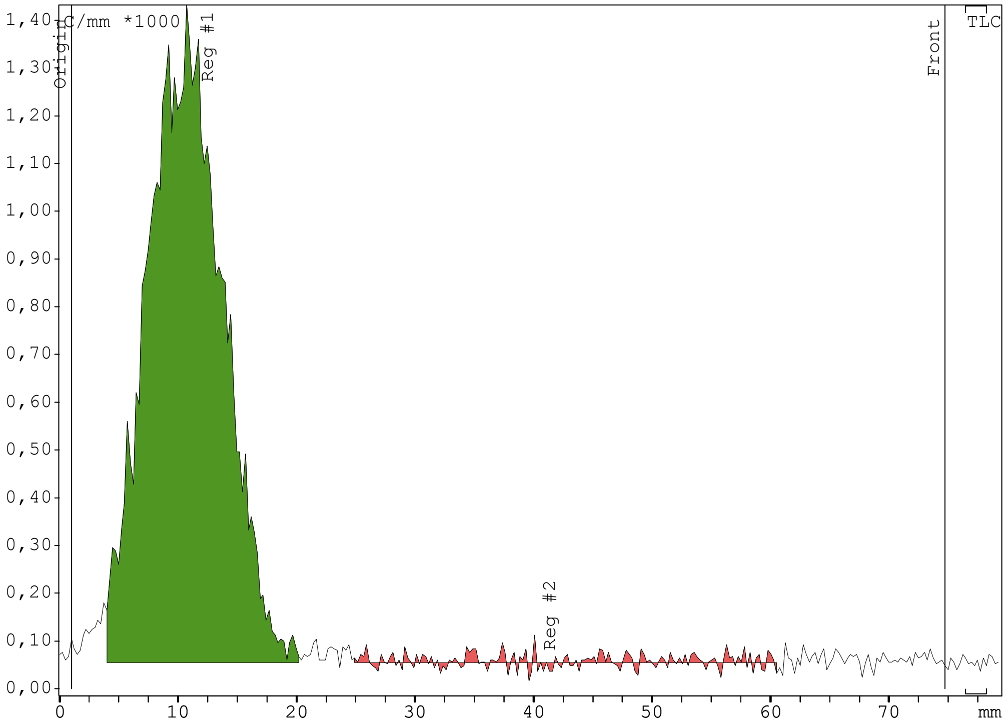


*Figure S8. Radio-iTLC chromatogram. Radiochemical yield of [$^{99m}$Tc]Tc-4Ac after incubation with 1000-fold molar excess of histidine for 4 h (stability test)*

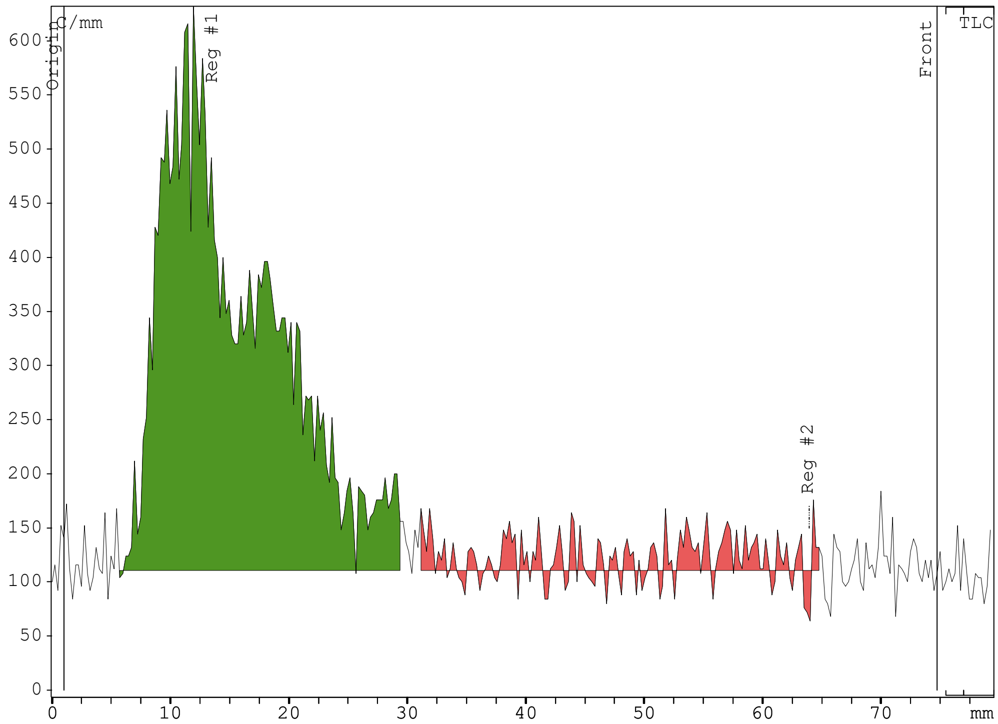


*Figure S9. Radio-iTLC chromatogram. Radiochemical yield of [$^{99m}$Tc]Tc-4Ac3N after incubation with 1000-fold molar excess of histidine for 4 h (stability test)*

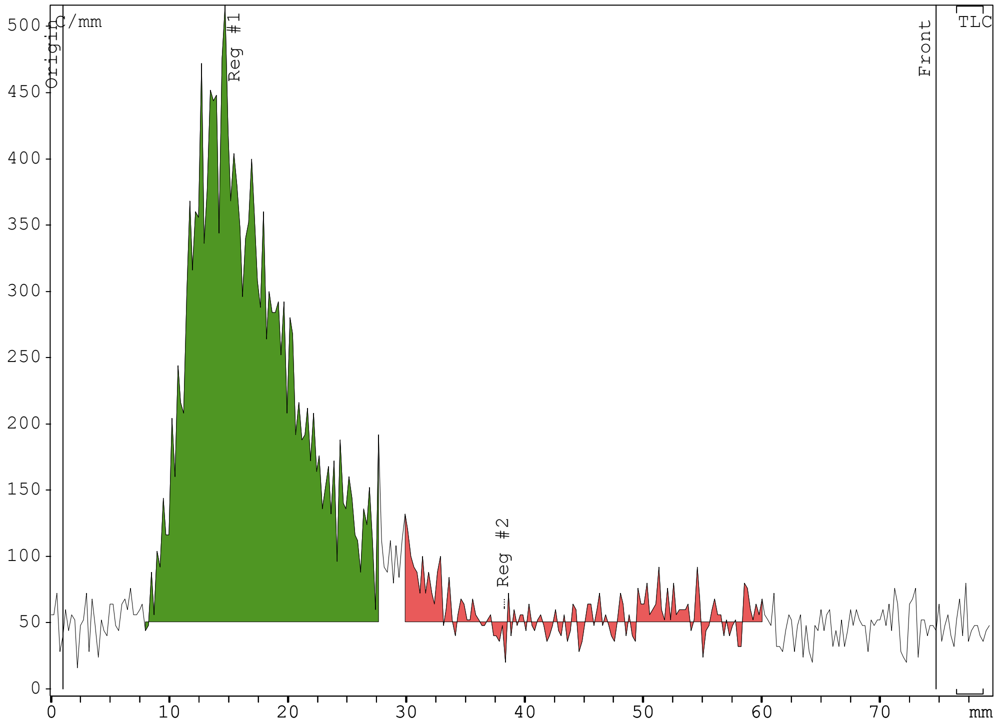


*Figure S10. Radio-iTLC chromatogram. Radiochemical yield of [$^{99m}$Tc]Tc-4Ac3N after incubation with PBS for 4 h (stability test)*

**Biodistribution of [99mTc]Tc(CO)3-4Ac3N**

Table S1. The dose-dependent biodistribution of $[^{99m}Tc]Tc(CO)_3$-4Ac3N in Nu/j mice bearing epidermoid carcinoma (A-431) xenografts at injected doses of 1.2 mg/kg, 6 mg/kg, 12 mg/kg 2 h post-injection. The uptake is displayed as average %ID/g ± SD, except for the GI tract and body, where it is presented as average %ID/sample ± SD.

| Organs | 1.2 mg/kg | 6 mg/kg | 12 mg/kg |
|---|---|---|---|
| **Blood** | 8.9 ± 1.1 | 8.5 ± 1.3 | 10.4 ± 1.2 |
| **Salivary glands** | 2.2 ± 0.3[a] | 3.0 ± 0.4 | 4.3 ± 1.2 |
| **Brain** | 0.5 ± 0.2[b] | 1.3 ± 0.2 | 1.0 ± 0.6 |
| **Heart** | 3.6 ± 1.6 | 3.3 ± 0.4 | 5.9 ± 2.3 |
| **Lung** | 4.4 ± 1.5 | 4.3 ± 0.6 | 6.7 ± 1.8 |
| **Liver** | 8.2 ± 1.2[a] | 8.9 ± 0.6 | 10.5 ± 0.8 |
| **Spleen** | 2.1 ± 0.8[a] | 3.7 ± 0.5 | 4.7 ± 1.4 |
| **Pancreas** | 1.4 ± 0.6 | 2.2 ± 0.4 | 6.6 ± 4.6 |
| **Small intestine** | 3.5 ± 0.9[a] | 4.5 ± 1.1[c] | 10.6 ± 4.7 |
| **Large intestine** | 12.7 ± 14.4 | 27.8 ± 24.9 | 20.5 ± 28.3 |
| **Stomach** | 2.0 ± 0.2[a] | 2.7 ± 0.6[c] | 5.0 ± 1.7 |
| **Kidney** | 5.7 ± 1.6 | 4.7 ± 1.0 | 5.9 ± 1.4 |
| **Muscle** | 0.7 ± 0.1 | 0.8 ± 0.1 | 1.1 ± 0.5 |
| **Bone** | 4.8 ± 7.4 | 1.8 ± 0.4 | 4.6 ± 2.0 |
| **Skin** | 1.8 ± 0.3 | 1.7 ± 0.1 | 3.4 ± 2.4 |
| **Fat** | 1.1 ± 1.0 | 0.7 ± 0.3 | 2.1 ± 1.4 |
| **Tumor** | 3.2 ± 2.8 | 1.8 ± 0.5 | 3.9 ± 1.4 |
| **GI** | 33.0 ± 7.0 | 38.0 ± 4.0 | 34.7 ± 1.3 |
| **BODY** | 16.2 ± 2.8 | 17.3 ± 1.5 | 19.0 ± 2.0 |

[a] – significant differences between $[^{99m}Tc]Tc(CO)_3$-4Ac3N 1.2 mg/kg & $[^{99m}Tc]Tc(CO)_3$-4Ac3N 12 mg/kg
[b] – significant differences between $[^{99m}Tc]Tc(CO)_3$-4Ac3N 1.2 mg/kg & $[^{99m}Tc]Tc(CO)_3$-4Ac3N 6 mg/kg
[c] – significant differences between $[^{99m}Tc]Tc(CO)_3$-4Ac3N 6 mg/kg & $[^{99m}Tc]Tc(CO)_3$-4Ac3N 12 mg/kg

Table S2. Biodistribution of [$^{99m}$Tc]Tc(CO)$_3$-4Ac3N in Nu/J mice with epidermoid carcinoma (A-431), prostate cancer (PC-3) and ovarian adenocarcinoma (SKOV-3) xenografts 2 h post-injection. The uptake is displayed as average %ID/g ± SD, except for the GI tract and body, where it is presented as average %ID/sample ± SD.

| **Organs** | **A-431** | **SKOV-3** | **PC-3** |
|---|---|---|---|
| **Blood** | 8.5 ± 1.3 | 7.5 ± 1.4 | 7.2 ± 1.3 |
| **Salivary glands** | 3.0 ± 0.4 [a,b] | 1.6 ± 0.4 | 1.6 ± 0.1 |
| **Brain** | 1.3 ± 0.2 [a,b] | 0.2 ± 0.1 | 0.2 ± 0.1 |
| **Heart** | 3.3 ± 0.4 [a,b] | 2.4 ± 0.5 | 2.4 ± 0.4 |
| **Lung** | 4.3 ± 0.6 | 3.9 ± 1.1 | 3.4 ± 0.6 |
| **Liver** | 8.9 ± 0.6 | 8.6 ± 2.2 | 8.4 ± 1.5 |
| **Spleen** | 3.7 ± 0.5 [a,b] | 2.0 ± 0.4 | 1.7 ± 0.1 |
| **Pancreas** | 2.2 ± 0.4 [a,b] | 1.0 ± 0.2 | 1.1 ± 0.3 |
| **Small intestine** | 4.5 ± 1.1 | 3.7 ± 1.6 | 3.6 ± 0.9 |
| **Large intestine** | 28.0 ± 25.0 | 3.1 ± 2.4 | 1.0 ± 0.4 |
| **Stomach** | 2.7 ± 0.6 | 2.3 ± 1.2 | 1.2 ± 0.1 |
| **Kidney** | 4.7 ± 1.0 | 4.0 ± 1.3 | 5.3 ± 0.4 |
| **Muscle** | 0.8 ± 0.1 [a,b] | 0.6 ± 0.1 | 0.5 ± 0.1 |
| **Bone** | 1.8 ± 0.4 [a,b] | 1.2 ± 0.2 | 0.9 ± 0.2 |
| **Skin** | 1.7 ± 0.1 | 1.5 ± 0.5 | 1.3 ± 0.2 |
| **Fat** | 0.7 ± 0.3 | 0.6 ± 0.3 | 0.6 ± 0.4 |
| **Tumor** | 1.8 ± 0.5 | 2.7 ± 0.7 | 2.3 ± 0.7 |
| **GI** | 38.0 ± 4.0 | 36.0 ± 6.0 | 38.0 ± 4.0 |
| **BODY** | 17.3 ± 1.5 | 15.0 ± 4.0 | 16.0 ± 2.0 |

[a] – significant differences between A-431 & SKOV-3
[b] – significant differences between A-431 & PC-3
[c] – significant differences between SKOV-3 & PC-3

Table S3. The biodistribution of $[^{99m}Tc]Tc(CO)_3$-4Ac3N in Nu/j mice bearing prostate cancer (PC-3) xenografts at 1, 3, 6, 24 and 48 h post-injection. The uptake is displayed as average %ID/g ± SD, except for the GI tract and body, where it is presented as average %ID/sample ± SD.

| **Organs** | **1h** | **3 h** | **6 h** | **24 h** | **48 h** |
|---|---|---|---|---|---|
| **Blood** | 10.8 ± 1.4[b,c,d] | 7.5 ± 2.8[f,g] | 5.8 ± 1.9[k,l] | 1.4 ± 0.1 | 0.6 ± 0.3 |
| **Salivary glands** | 2.4 ± 0.6[c,d] | 1.9 ± 0.3[f,g] | 1.7 ± 0.5[l] | 0.76 ± 0.12 | 0.54 ± 0.03 |
| **Brain** | 0.3 ± 0.1[c] | 0.2 ± 0.1[f,g] | 0.2 ± 0.1[k,l] | 0.06 ± 0.02 | 0.08 ± 0.03 |
| **Heart** | 2.9 ± 0.5[b,c] | 2.2 ± 0.3 | 1.9 ± 0.3 | 0.8 ± 0.1 | 0.72 ± 0.07 |
| **Lung** | 5.3 ± 2.4[c,d] | 3.2 ± 0.4 | 2.9 ± 0.4 | 1.2 ± 0.2 | 1.1 ± 0.1 |
| **Liver** | 12.1 ± 1.5[c,d] | 10.1 ± 0.5[g] | 10.64 ± 1.04[k,l] | 7.8 ± 1.3 | 6.5 ± 0.6 |
| **Spleen** | 2.2 ± 0.1[c,d] | 1.9 ± 0.3[g] | 1.7 ± 0.6 | 1.3 ± 0.1 | 1.0 ± 0.3 |
| **Pancreas** | 1.1 ± 0.1[c,d] | 0.9 ± 0.4[g] | 0.9 ± 0.31 | 0.5 ± 0.1 | 0.34 ± 0.05 |
| **Small intestine** | 4.7 ± 1.7[a,b,c,d] | 2.2 ± 0.9 | 1.8 ± 0.3 | 0.98 ± 0.36 | 0.74 ± 0.14 |
| **Large intestine** | 0.8 ± 0.1[b] | 8.1 ± 4.0 | 25.0 ± 20.3[k,l] | 1.5 ± 0.6 | 0.57 ± 0.05 |
| **Stomach** | 4.0 ± 2.2[c,d] | 3.5 ± 1.2[g] | 3.5 ± 0.9[l] | 0.98 ± 0.24 | 0.65 ± 0.24 |
| **Kidney** | 6.1 ± 0.7[b] | 7.3 ± 0.7[g] | 8.3 ± 1.1[l] | 6.5 ± 1.3 | 5.16 ± 0.18 |
| **Muscle** | 0.50 ± 0.04[a,b,c,d] | 0.39 ± 0.05[f,g] | 0.34 ± 0.03[k,l] | 0.18 ± 0.01 | 0.17 ± 0.02 |
| **Bone** | 1.1 ± 0.5 | 1.1 ± 0.2 | 0.98 ± 0.15 | 0.5 ± 0.3 | 0.55 ± 0.14 |
| **Skin** | 1.0 ± 0.1 | 1.0 ± 0.1 | 1.1 ± 0.1 | 0.7 ± 0.1 | 0.7 ± 0.3 |
| **Fat** | 0.4 ± 0.2 | 0.6 ± 0.3 | 0.5 ± 0.3 | 0.19 ± 0.07 | 0.74 ± 0.77 |
| **Tumor** | **3.2 ± 0.7** | **2.5 ± 1.3** | **3.2 ± 1.6** | **2.6 ± 0.8**[z] | **1.2 ± 0.15** |
| **GI** | 25.8 ± 5.5[c,d] | 31.5 ± 4.8[e,f,g] | 18.3 ± 5.2[k,l] | 1.7 ± 0.6 | 0.9 ± 0.1 |
| **BODY** | 17.6 ± 2.7[b,c] | 14.5 ± 0.8[f,g] | 12.2 ± 2.9[k,l] | 6.5 ± 1.2 | 5.1 ± 0.4 |

[a] – significant differences between $[^{99m}Tc]Tc(CO)_3$-4Ac3N 1 h & $[^{99m}Tc]Tc(CO)_3$-4Ac3N 3 h
[b] – significant differences between $[^{99m}Tc]Tc(CO)_3$-4Ac3N 1 h & $[^{99m}Tc]Tc(CO)_3$-4Ac3N 6 h
[c] – significant differences between $[^{99m}Tc]Tc(CO)_3$-4Ac3N 1 h & $[^{99m}Tc]Tc(CO)_3$-4Ac3N 24 h
[d] – significant differences between $[^{99m}Tc]Tc(CO)_3$-4Ac3N 1 h & $[^{99m}Tc]Tc(CO)_3$-4Ac3N 48 h
[e] – significant differences between $[^{99m}Tc]Tc(CO)_3$-4Ac3N 3 h & $[^{99m}Tc]Tc(CO)_3$-4Ac3N 6 h
[f] – significant differences between $[^{99m}Tc]Tc(CO)_3$-4Ac3N 3 h & $[^{99m}Tc]Tc(CO)_3$-4Ac3N 24 h
[g] – significant differences between $[^{99m}Tc]Tc(CO)_3$-4Ac3N 3 h & $[^{99m}Tc]Tc(CO)_3$-4Ac3N 48 h
[k] – significant differences between $[^{99m}Tc]Tc(CO)_3$-4Ac3N 6 h & $[^{99m}Tc]Tc(CO)_3$-4Ac3N 24 h
[l] – significant differences between $[^{99m}Tc]Tc(CO)_3$-4Ac3N 6 h & $[^{99m}Tc]Tc(CO)_3$-4Ac3N 48 h
[z] – significant differences between $[^{99m}Tc]Tc(CO)_3$-4Ac3N 24 h & $[^{99m}Tc]Tc(CO)_3$-4Ac3N 48 h

Table S4. Comparison of organ-to-blood ratios from the biodistribution of [$^{99m}$Tc]Tc(CO)$_3$-4Ac3N in Nu/j mice bearing prostate cancer (PC-3) xenografts 1, 3, 6, 24 and 48 h post-injection.

| Organs | $^{99m}$Tc-4Ac3N 1h | $^{99m}$Tc-4Ac3N 3 h | $^{99m}$Tc-4Ac3N 6 h | $^{99m}$Tc-4Ac3N 24 h | $^{99m}$Tc-4Ac3N 48 h |
|---|---|---|---|---|---|
| **Salivary glands** | 0.23$^{d}$ | 0.28$^{g}$ | 0.31 | 0.53$^{z}$ | 1.07 |
| **Brain** | 0.03$^{d}$ | 0.03$^{g}$ | 0.04$^{l}$ | 0.04$^{z}$ | 0.17 |
| **Heart** | 0.27$^{d}$ | 0.32$^{g}$ | 0.34$^{l}$ | 0.56$^{z}$ | 1.43 |
| **Lung** | 0.51$^{d}$ | 0.46$^{g}$ | 0.53$^{l}$ | 0.85$^{z}$ | 2.08 |
| **Liver** | 1.13$^{d}$ | 1.47$^{g}$ | 1.97$^{l}$ | 5.42$^{z}$ | 12.82 |
| **Spleen** | 0.21$^{c,d}$ | 0.26$^{f,g}$ | 0.33$^{l}$ | 0.87$^{z}$ | 1.83 |
| **Pancreas** | 0.10$^{d}$ | 0.12$^{g}$ | 0.17$^{l}$ | 0.31 | 0.68 |
| **Small intestine** | 0.44$^{d}$ | 0.30$^{g}$ | 0.33$^{l}$ | 0.69 | 1.48 |
| **Large intestine** | 0.07$^{b}$ | 1.12$^{e}$ | 3.91$^{k,l}$ | 1.01 | 1.17 |
| **Stomach** | 0.38 | 0.48 | 0.65 | 0.68 | 1.39 |
| **Kidney** | 0.58$^{d}$ | 1.07$^{g}$ | 1.51$^{l}$ | 4.53$^{z}$ | 10.44 |
| **Muscle** | 0.05$^{d}$ | 0.06$^{g}$ | 0.06$^{l}$ | 0.12$^{z}$ | 0.34 |
| **Bone** | 0.11$^{d}$ | 0.16$^{g}$ | 0.19$^{l}$ | 0.35$^{z}$ | 1.10 |
| **Skin** | 0.10$^{d}$ | 0.15$^{g}$ | 0.19$^{l}$ | 0.49 | 1.61 |
| **Fat** | 0.04 | 0.09 | 0.07 | 0.13 | 1.78 |
| **Tumor** | 0.31$^{c,d}$ | 0.35$^{f,g}$ | 0.62$^{l}$ | 1.78 | 2.41 |
| **GI** | 2.46 | 4.52$^{f,g}$ | 3.38 | 1.20 | 1.84 |
| **BODY** | 1.65$^{d}$ | 2.10$^{g}$ | 2.21$^{l}$ | 4.53$^{z}$ | 10.42 |

$^{a}$ – significant differences between [$^{99m}$Tc]Tc(CO)$_3$-4Ac3N 1 h & [$^{99m}$Tc]Tc(CO)$_3$-4Ac3N 3 h
$^{b}$ – significant differences between [$^{99m}$Tc]Tc(CO)$_3$-4Ac3N 1 h & [$^{99m}$Tc]Tc(CO)$_3$-4Ac3N 6 h
$^{c}$ – significant differences between [$^{99m}$Tc]Tc(CO)$_3$-4Ac3N 1 h & [$^{99m}$Tc]Tc(CO)$_3$-4Ac3N 24 h
$^{d}$ – significant differences between [$^{99m}$Tc]Tc(CO)$_3$-4Ac3N 1 h & [$^{99m}$Tc]Tc(CO)$_3$-4Ac3N 48 h
$^{e}$ – significant differences between [$^{99m}$Tc]Tc(CO)$_3$-4Ac3N 3 h & [$^{99m}$Tc]Tc(CO)$_3$-4Ac3N 6 h
$^{f}$ – significant differences between [$^{99m}$Tc]Tc(CO)$_3$-4Ac3N 3 h & [$^{99m}$Tc]Tc(CO)$_3$-4Ac3N 24 h
$^{g}$ – significant differences between [$^{99m}$Tc]Tc(CO)$_3$-4Ac3N 3 h & [$^{99m}$Tc]Tc(CO)$_3$-4Ac3N 48 h
$^{k}$ – significant differences between [$^{99m}$Tc]Tc(CO)$_3$-4Ac3N 6 h & [$^{99m}$Tc]Tc(CO)$_3$-4Ac3N 24 h
$^{l}$ – significant differences between [$^{99m}$Tc]Tc(CO)$_3$-4Ac3N 6 h & [$^{99m}$Tc]Tc(CO)$_3$-4Ac3N 48 h
$^{z}$ – significant differences between [$^{99m}$Tc]Tc(CO)$_3$-4Ac3N 24 h & [$^{99m}$Tc]Tc(CO)$_3$-4Ac3N 48 h

Table S5. Comparison of tumor-to-organ ratios of $[^{99m}Tc]Tc(CO)_3$-4Ac3N in Nu/j mice bearing prostate cancer (PC-3) xenografts at 1, 3, 6, 24 and 48 h post-injection.

| Organs | 1h | 3 h | 6 h | 24 h | 48 h |
|---|---|---|---|---|---|
| **Blood** | 0.31 ± 0.08[c,d] | 0.6 ± 0.5[f,g] | 0.6 ± 0.4[l] | 1.8 ± 0.5 | 2.41 ± 1.01 |
| **Salivary glands** | 1.4 ± 0.3[c] | 1.2 ± 0.8[f,g] | 1.9 ± 0.6[k] | 3.4 ± 0.8 | 2.3 ± 0.2 |
| **Brain** | 10.7 ± 2.3[c] | 13.5 ± 12.7[f] | 18.4 ± 11.6[k] | 44.4 ± 11.6[z] | 15.25 ± 3.01 |
| **Heart** | 1.1 ± 0.3[c] | 1.0 ± 0.8[f] | 1.7 ± 0.7[k] | 3.1 ± 0.7[z] | 1.7 ± 0.1 |
| **Lung** | 0.7 ± 0.2[c] | 0.7 ± 0.5[f] | 1.09 ± 0.5[k] | 2.1± 0.4[z] | 1.2 ± 0.2 |
| **Liver** | 0.27 ± 0.04 | 0.73 ± 0.96 | 0.3 ± 0.1 | 0.3 ± 0.1 | 0.19 ± 0.01 |
| **Spleen** | 1.5 ± 0.3 | 1.3 ± 0.7 | 1.96 ± 0.96 | 2.1 ± 0.7 | 1.3 ± 0.4 |
| **Pancreas** | 3.1 ± 0.6 | 3.0 ± 2.4 | 3.47 ± 0.66 | 5.7 ± 1.4 | 3.8 ± 0.9 |
| **Small intestine** | 0.7 ± 0.1c | 1.4 ± 0.8 | 1.74 ± 0.7 | 2.96 ± 1.69 | 1.7 ± 0.5 |
| **Large intestine** | 4.3 ± 0.8[a,b,c,d] | 0.33 ± 0.37[f,g] | 0.22 ± 0.2[k,l] | 1.9 ± 0.6 | 2.2 ± 0.4 |
| **Stomach** | 0.97 ± 0.52[c] | 0.7 ± 0.6[f] | 0.88 ± 0.27[k] | 2.6 ± 0.7 | 2.1 ± 0.8 |
| **Kidney** | 0.5 ± 0.1 | 0.3 ± 0.3 | 0.39 ± 0.19 | 0.4 ± 0.1 | 0.24 ± 0.03 |
| **Muscle** | 6.5 ± 1.7 | 5.4 ± 5.6 | 9.28 ± 4.16 | 14.8 ± 5.3 | 6.9 ± 0.5 |
| **Bone** | 3.7 ± 2.7 | 1.8 ± 1.8 | 3.12 ± 1.09 | 15.7 ± 3.8 | 2.3 ± 0.6 |
| **Skin** | 3.2 ± 0.6 | 2.1 ± 2.1 | 3.09 ± 1.86 | 3.8 ± 1.7 | 1.95 ± 0.79 |
| **Fat** | 9.6 ± 4.4 | 5.6 ± 7.6 | 9.8 ± 7.82 | 14.96 ± 7.27 | 2.8 ± 1.6 |

[a] – significant differences between $[^{99m}Tc]Tc(CO)_3$-4Ac3N 1 h & $[^{99m}Tc]Tc(CO)_3$-4Ac3N 3 h
[b] – significant differences between $[^{99m}Tc]Tc(CO)_3$-4Ac3N 1 h & $[^{99m}Tc]Tc(CO)_3$-4Ac3N 6 h
[c] – significant differences between $[^{99m}Tc]Tc(CO)_3$-4Ac3N 1 h & $[^{99m}Tc]Tc(CO)_3$-4Ac3N 24 h
[d] – significant differences between $[^{99m}Tc]Tc(CO)_3$-4Ac3N 1 h & $[^{99m}Tc]Tc(CO)_3$-4Ac3N 48 h
[e] – significant differences between $[^{99m}Tc]Tc(CO)_3$-4Ac3N 3 h & $[^{99m}Tc]Tc(CO)_3$-4Ac3N 6 h
[f] – significant differences between $[^{99m}Tc]Tc(CO)_3$-4Ac3N 3 h & $[^{99m}Tc]Tc(CO)_3$-4Ac3N 24 h
[g] – significant differences between $[^{99m}Tc]Tc(CO)_3$-4Ac3N 3 h & $[^{99m}Tc]Tc(CO)_3$-4Ac3N 48 h
[k] – significant differences between $[^{99m}Tc]Tc(CO)_3$-4Ac3N 6 h & $[^{99m}Tc]Tc(CO)_3$-4Ac3N 24 h
[l] – significant differences between $[^{99m}Tc]Tc(CO)_3$-4Ac3N 6 h & $[^{99m}Tc]Tc(CO)_3$-4Ac3N 48 h
z- significant differences between $[^{99m}Tc]Tc(CO)_3$-4Ac3N 24 h & $[^{99m}Tc]Tc(CO)_3$-4Ac3N 48 h
